# Development and Functional Evaluation of a Pharmabiotic Food Supplement Containing Microencapsulated *Lactiplantibacillus pentosus* for Oral Microbiome Health

**DOI:** 10.1002/fsn3.72243

**Published:** 2026-08-09

**Authors:** Pervin Soyer, A. Alper Öztürk

**Affiliations:** ^1^ Faculty of Pharmacy, Department of Pharmaceutical Microbiology Anadolu University Eskisehir Turkiye; ^2^ Faculty of Pharmacy, Department of Pharmaceutical Technology Anadolu University Eskisehir Turkiye

**Keywords:** *Lactiplantibacillus pentosus*, microencapsulation, oral spray, pharmabiotic

## Abstract

Oral microbiome dysbiosis is associated with biofilm‐related diseases, including dental caries and periodontal infections. This study aimed to develop and evaluate a pharmabiotic oral spray containing microencapsulated Lactiplantibacillus pentosus, sodium hyaluronate, and licorice ( *Glycyrrhiza glabra* ) extract to support oral microbial homeostasis. *L. pentosus* was microencapsulated by freeze‐drying using maltodextrin and gum arabic as wall materials. The microencapsulation process resulted in an encapsulation efficiency of 94.74% ± 1.12% and a post‐encapsulation viability of 8.17 ± 0.05 log CFU/mL. Four oral spray formulations (F1–F4) were prepared and evaluated for antimicrobial, antibiofilm, antioxidant, physicochemical, microbiological, and stability properties. All formulations exhibited antibacterial activity against Gram‐positive oral pathogens, particularly *Streptococcus mutans* and *Staphylococcus aureus* , while no inhibitory activity was observed against *Pseudomonas aeruginosa* or *Candida* species. Antibiofilm analysis demonstrated substantial inhibition, ranging from 56.17% ± 0.91% to 70.99% ± 1.32% against *S. mutans* and from 72.42% ± 0.56% to 87.03% ± 1.76% against *S. aureus* . Antioxidant assays (DPPH, ABTS, and ORAC) confirmed moderate and consistent antioxidant activity across all formulations. During 3 months of storage, probiotic viability remained above 10^8^ CFU/mL in most formulations. Viability losses under refrigerated conditions were limited to approximately 0.38–0.50 log CFU/mL, while formulation F2 retained 8.42 ± 0.14 log CFU/mL under accelerated conditions. Among the tested formulations, F1 exhibited the most favorable overall physicochemical characteristics and stability profile. These findings demonstrate that the developed oral spray represents a promising pharmabiotic delivery system combining probiotic viability, antibiofilm activity, and antioxidant potential for supporting oral health and microbial balance.

## Graphical abstract

Development of a multifunctional pharmabiotic oral spray containing microencapsulated *Lactiplantibacillus pentosus*, hyaluronic acid, and *Glycyrrhiza glabra* extract. The formulation synergistically suppresses oral biofilms while exhibiting antimicrobial and antioxidant activities, supporting oral microbiome homeostasis.

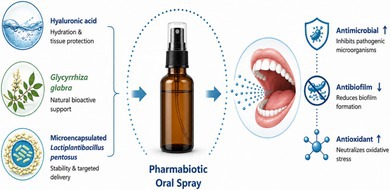

## 1 Introduction

The human body is made up of several eco‐systems that are composed of micro‐eco‐systems, just like the earth. Every microbiota has its own habitat, and the microorganisms that live there have a harmonious balance (homeostasis) of temperature, pH, nutrients, and byproducts that are either symbiotically or synergistically distributed (Bugshan et al. 2017). Normally found in the mouth, the oral microbiome is a dynamic and complex collection of microorganisms that support human health through a variety of mechanisms, such as immunological optimization and the exclusion of pathogenic microbes (Kilian et al. 2016). The teeth, gingival sulcus, connected gingiva, tongue, cheek, lip, hard palate, and soft palate are some of the unique microbial habitats found in the oral cavity, also known as the mouth. The most diverse microbiota found in the oral microbiome is mostly composed of 500–1000 species of bacteria, with a variety of fungi, viruses, protozoa, and archaea following (Li et al. 2022). Under healthy conditions, these microorganisms exist in a dynamic ecological balance that contributes to oral and systemic health. Disruption of this balance may lead to oral dysbiosis, biofilm accumulation, and the development of oral diseases such as dental caries and periodontitis.

Modification brought on by systemic or local variables will result in imbalance or “dysbiosis,” which will encourage the growth of potentially harmful microbes, such as persistent inflammation (Bugshan et al. 2017). It has been demonstrated that oral dysbiosis, in which the relative abundance of microbiota at a specific place or organ varies dramatically to modify the physiological functioning of the microbiome, is essential to the pathogenesis of a number of clinical disorders on both a local and systemic level (Hajishengallis et al. 2011; Tsai et al. 2018). Many oral and systemic disorders, such as dental caries (Di Stefano et al. 2022), periodontitis (Costalonga and Herzberg 2014), oral and other cancers (Gholizadeh et al. 2016), cardiovascular disease (Tonelli et al. 2023), and diabetes (Long et al. 2017), can be attributed to dysbiosis of the oral microbiome. Biofilms are organized microbial communities that are adhered to inert or living surfaces and are made up of bacteria and fungus embedded in an extracellular polysaccharide matrix that they manufacture on their own. Instead of existing as free‐floating cells, bacteria in the oral cavity are primarily found in this organized form. As early colonists, *Streptococcus* species influence the makeup of later microbial communities through their first adherence. Dysbiosis, which is defined by an overabundance of pathogenic species, greater biofilm durability, and improved resistance to antimicrobial treatments, results from disruption of this balanced framework. In addition to providing additional protection, the extracellular polysaccharide matrix promotes nutrition flow and intercellular communication, which aids in the persistence of pathogens and the development of disease (Fux et al. 2003; Santacroce et al. 2023).

There is a correlation between a rise in harmful bacteria and a fall in helpful bacteria. Prebiotics, probiotics, and antimicrobial agents are examples of interventions to restore microbial health and balance in the oral microbiome that have been the subject of extensive research as potential therapeutic approaches for treating and preventing oral dysbiosis and its associated diseases (Hill et al. 2014; Maier 2023; Salminen et al. 2021). Live bacteria known as probiotics have positive effects on the host (Szajewska et al. 2020). Probiotics should be safe to use, according to guidelines from the World Health Organization and the Food and Agriculture Organization. No hemolytic activity, no formation of D‐lactate, no production of biogenetic amines, and no transferable antibiotic resistance are frequently included in safety evaluations (Huys et al. 2013). The majority of probiotics belong to the *Bifidobacterium* or *Lactobacillus* genera. To hinder pathogenicity, *Lactobacillus* species, in particular, constitute a significant portion of the oral microbiota (Wong et al. 2013). Thus far, an effective oral probiotic has been identified by assessing its effectiveness in vitro, which includes the production of antimicrobial components (hydrogen peroxide, lactic acid, and bacteriocins) that can prevent the growth of oral pathogens, high tolerance to environmental stress (acid and lysozyme tolerance) (Kõll et al. 2008), no propagating antibiotic resistance, good auto‐aggregation and co‐aggregation ability, and adhesion to oral epithelial cells (Terai et al. 2015). Lactic acid bacteria (LAB) have the potential to improve dental health, according to recent research (Haukioja 2010). Lactic acid bacteria have been produced to protect and improve oral health and are found in a variety of pharmaceutical formulations, including lozenges, oral sprays, and tablets (Sanders et al. 2019). In the oral cavity, probiotic‐containing oral sprays in particular have beneficial effects on lowering the risk of dental caries and other conditions. The physicochemical technique of probiotic microencapsulation creates spherical particles with thin, robust, semipermeable membranes that range in diameter from nanometers to millimeters by trapping a probiotic within an appropriate material in pharmaceutical formulations (Frakolaki et al. 2021). By encapsulating live organisms from external stresses including temperature, oxygen, and moisture, microencapsulation significantly contributes to the stability of probiotic formulations. Preventing the loss of cell viability during storage is crucial because the shelf life of probiotic products is mostly dependent on keeping a sufficient quantity of live bacteria. Microencapsulation improves long‐term stability and guarantees maintained probiotic efficacy over the course of the product's shelf life by offering a physical barrier and controlled release characteristics (Amaya Cano et al. 2021). Although several commercially available oral probiotic products contain well‐established strains such as *Lacticaseibacillus rhamnosus*, *Limosilactobacillus reuteri*, and *Streptococcus salivarius* , most are marketed as tablets, lozenges, capsules, or chewable dosage forms and are primarily designed to deliver probiotic microorganisms alone. Oral spray formulations offer several advantages, including ease of administration, uniform distribution within the oral cavity, and prolonged contact with oral tissues. However, maintaining probiotic viability in liquid oral formulations remains a major technological challenge. Microencapsulation has emerged as an effective strategy for improving probiotic stability, protecting cells against environmental stress, and enhancing storage performance.

Pharmabiotics are probiotics produced in pharmaceutical forms and used in the treatment of diseases and various clinical conditions by modulating human physiochemistry. Pharmabiotics can be administered through various routes, including oral (Baral et al. 2021), topical (Rather et al. 2018), vaginal (Vigani et al. 2019), rectal, nasal, and even ocular routes (Jung et al. 2019). For oral administration, different dosage forms such as sprays, tablets, capsules, granules, pellets, and powders are available. In contrast to conventional probiotic oral products, the present study aimed to develop a multifunctional pharmabiotic oral spray combining microencapsulated *Lactiplantibacillus pentosus*, sodium hyaluronate, and licorice ( *Glycyrrhiza glabra* ) extract within a single delivery platform. To improve probiotic stability, preserve cell viability while in storage, and guarantee controlled release when applied to the oral cavity, microencapsulation was used. In order to enable prolonged local distribution and activity of the encapsulated probiotic cells, sodium hyaluronate was added as a mucoadhesive polymer to extend the formulation's residence time on oral mucosal surfaces. Because licorice root ( *Glycyrrhiza glabra* ) extract has proven antibacterial and antibiofilm qualities, it was used to help suppress oral infections and disrupt the production of biofilms. The developed formulation was designed as a multifunctional oral delivery system in which sodium hyaluronate enhances mucosal adhesion and retention, licorice ( *Glycyrrhiza glabra* ) extract provides antibacterial, antibiofilm, and antioxidant bioactivities, and microencapsulated *Lactiplantibacillus pentosus* contributes to oral microbiota modulation and functional biological activity. The combination of these components was expected to exert complementary and potentially synergistic effects in supporting oral health. These ingredients work together to produce a stable, locally acting oral spray formulation that promotes oral health and microbial balance. To the best of our knowledge, studies investigating the combination of microencapsulated *Lactiplantibacillus pentosus*, sodium hyaluronate, and licorice ( *Glycyrrhiza glabra* ) extract within a probiotic oral spray platform remain limited. Therefore, the present study addresses an important knowledge gap by evaluating not only probiotic stability and viability but also the antimicrobial, antibiofilm, and antioxidant performance of a multifunctional oral pharmabiotic system. The study especially aimed to include the stabilized probiotic in a mucoadhesive oral spray system enhanced with bioactive plant extract and to improve probiotic stability through microencapsulation based on maltodextrin–gum arabic. Additionally, the produced formulations' biological performance was assessed in terms of their antioxidant, antimicrobial, and antibiofilm properties. In order to evaluate the formulations' potential as a stable and effective oral administration system, their physicochemical properties, microbiological safety, and probiotic viability were also evaluated.

## 2 Materials and Methods

The probiotic strain *Lactiplantibacillus pentosus* NRRL B‐227 was obtained from the ARS Culture Collection (Agricultural Research Service, Illinois, USA) and the German Collection of Microorganisms and Cell Cultures GmbH (DSMZ, Germany). As a reference strain maintained by internationally recognized culture repositories, *L. pentosus* NRRL B‐227 has been taxonomically authenticated and preserved under standardized quality‐control procedures. The strain was selected for this study due to its reported probiotic properties and its suitability for incorporation into probiotic‐based pharmaceutical formulations. Sodium hyaluronate (Sigma‐Aldrich, St. Louis, MO, USA), maltodextrin (Merck, Darmstadt, Germany), gum arabic (Merck, Darmstadt, Germany), glycerin (Sigma‐Aldrich, St. Louis, MO, USA), sorbitol solution (70%, w/w) (Merck, Darmstadt, Germany), xylitol (Sigma‐Aldrich, St. Louis, MO, USA), sodium bicarbonate (Merck, Darmstadt, Germany), sodium chloride (Merck, Darmstadt, Germany), sodium benzoate (Sigma‐Aldrich, St. Louis, MO, USA), potassium sorbate (Sigma‐Aldrich, St. Louis, MO, USA), ascorbic acid (Merck, Darmstadt, Germany), and licorice ( *Glycyrrhiza glabra* ) extract obtained from a local herbal supplier in Türkiye were used for formulation development.

### 2.1 Microencapsulation of *Lactiplantibacillus pentosus*

**FIGURE 1:**
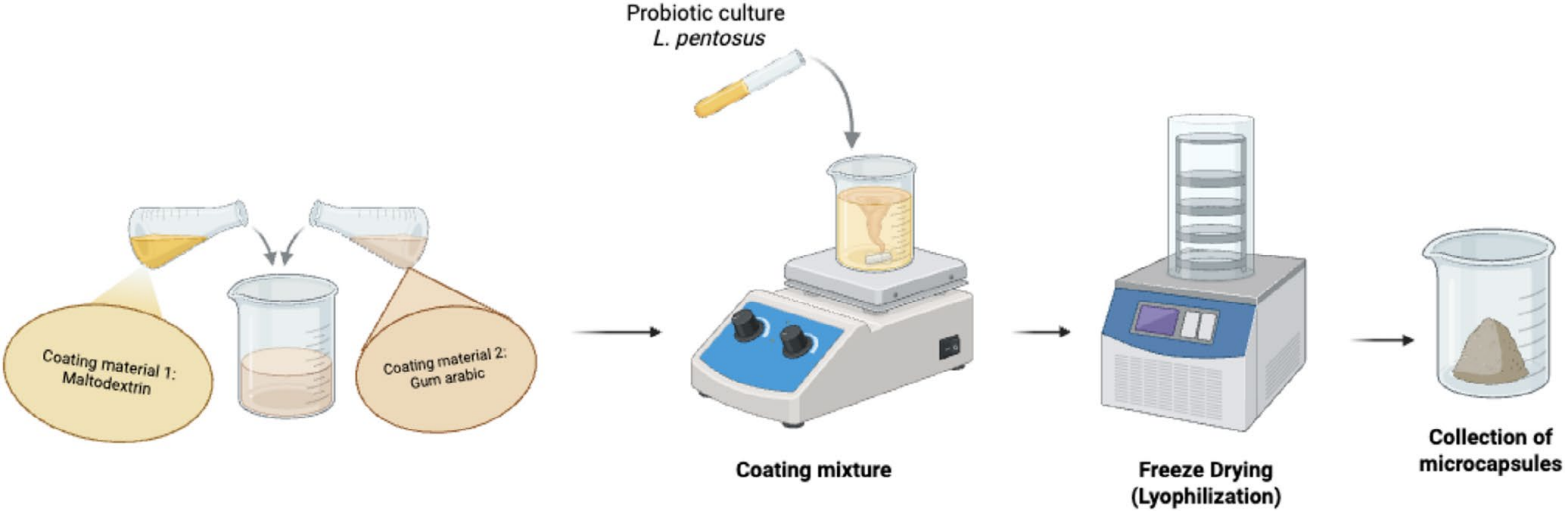
Flow diagram illustrating the microencapsulation process of *Lactiplantibacillus pentosus usi*ng maltodextrin and gum arabic as wall materials. The coating materials were dissolved in distilled water and mixed with the probiotic culture to form a homogeneous suspension. The mixture was subsequently freeze‐dried (lyophilized) to produce dry probiotic microcapsules, which were collected and stored in powder form for further formulation studies.

To enhance the stability and technological suitability of *Lactiplantibacillus pentosus* for oral spray formulation, the probiotic strain was microencapsulated using a freeze‐drying (lyophilization) technique with maltodextrin and gum arabic as wall materials. The activated bacterial culture was propagated in 50 mL MRS broth at 37°C for 24 h. Following incubation, cells were harvested by centrifugation at 2000 ***g*** for 10 min at 4°C. The resulting pellet was washed with 10 mL sterile peptone water and centrifuged again under the same conditions to remove residual medium components. The final pellet was resuspended and adjusted to approximately 10^9^ CFU/mL using a densitometer according to the McFarland turbidity standard (McFarland 4), which was used as the core material for encapsulation. For preparation of the wall matrix, maltodextrin (15%, w/v) and gum arabic (15%, w/v) were dissolved in distilled water to obtain a total solid content of 30% (w/v). The solution was stirred until complete dissolution was achieved and subjected to thermal treatment at 80°C for 30 min to ensure homogeneity and microbial safety. After cooling to room temperature, the probiotic suspension was incorporated into the polymer solution at a final culture concentration of 20% (v/v) and homogenized at 5000 rpm for 2 min to obtain a uniform encapsulation mixture. The prepared suspension was frozen at −20°C for 24 h to allow complete solidification and subsequently transferred to a lyophilizer operating at −85°C under a condenser pressure of 50–60 mTorr for 28 h. Following freeze‐drying, the resulting dry microencapsulated probiotic powders were collected and stored in sterile screw‐capped containers at 4°C until further formulation and analysis (Figure 1) (Moayyedi et al. 2018).

### 2.2 Viability Determination and Encapsulation Efficiency

The release of entrapped probiotic cells and determination of viable counts were performed (Chen et al. 2005). Briefly, 0.1 g of microencapsulated powder was accurately weighed and suspended in 10 mL of sterile 0.1 M potassium phosphate buffer (KH_2_PO_4_, pH 7.0). The suspension was homogenized for 15 min to ensure complete disruption of the polymeric matrix and release of the encapsulated bacterial cells. Following homogenization, serial dilutions were prepared using sterile peptone water, and appropriate dilutions were spread onto MRS agar plates. The plates were incubated at 37°C for 48 h, after which colonies were counted and results were expressed as CFU/mL of microencapsulated powder. Encapsulation efficiency (EE%) was calculated by comparing the viable cell count after encapsulation (N) with the initial viable cell count before encapsulation (N_0_) using the following equation (1):

(1) $$\mathrm{EE}\%=\frac{\log N}{\log N_{0}}\times 100$$

Following confirmation of sufficient probiotic viability and encapsulation efficiency, the microencapsulated *Lactiplantibacillus pentosus* was incorporated into the oral spray formulations.

### 2.3 Characterization of Microcapsules

#### 2.3.1 Morphological and Particle Size Analysis

The morphologies and particle size of the microcapsules and ratios were observed by using a scanning electron microscope (HITACHI‐TM3030 Plus Tabletop‐Microscope Desktop Electron Microscope). Imaging was performed under high vacuum at voltages between 5 and 15 kV. The samples were examined under 500×, 1000×, and 1500× magnifications.

#### 2.3.2 Moisture Content and Hygroscopicity Analysis

The average moisture content (wet basis %) of the freeze‐dried microencapsulated powder was measured gravimetrically. The 0.5 g sample was placed in aluminium pan and dried in a hot air oven at 105°C ± 2°C for 24 h. The sample was then removed and immediately weighed to limit water absorption from the atmosphere. The initial and final weights were used to calculate the wet basis moisture content. The experiments were carried out in triplicates and average values were taken.

The hygroscopicity of the microencapsulated probiotic powder was determined according to the procedure described by Fritzen‐Freire et al. (2013). More specifically, 1 g sample of the powder was placed in a desiccator equilibrated at 75% relative humidity containing a saturated sodium chloride (NaCl) solution at room temperature (Fritzen‐Freire et al. 2013). Samples were kept for 7 days and hygroscopicity was calculated gravimetrically according to the formula (2).

(2) Hygroscopicity%=𝑚𝑓−𝑚𝑖/𝑚𝑖×100%

*m*_*i*_ and *m*_*f*_ express the moisture of the samples prior to and after storage at 75% relative humidity.

#### 2.3.3 Evaluation of the Survival of Encapsulated Probiotics Exposed to Simulated Gastrointestinal Conditions

The encapsulated culture of *L. pentosus* was evaluated for viability under the simulated gastric and intestinal juice. Survival capacity of encapsulated probiotics during in vitro digestion process (gastrointestinal conditions) was determined by plate‐counting method with the samples of simulated gastric juice (GJ) and intestinal juice (IJ). To prepare the simulated gastric juice, 0.08 M HCl containing 0.2% (w/v) NaCl was adjusted to pH 2 and sterilized at 121°C for 20 min followed by adding 0.3% pepsin. Simulated intestinal juice was prepared by dissolving 0.6% bile salt in intestinal solution (6.5 g/L NaCl, 0.835 g/L KCl, 0.22 g/L CaCl_2_ and 1.386 g/L NaHCO_3_) pH 7.5 to final concentrations of 3 g/L and sterilized at 121°C for 20 min. To evaluate the gastrointestinal survival of microencapsulated probiotic cells fresh beads (1 g) were added to test tubes containing 10 mL of prewarmed (37°C) simulated gastric and intestinal juice and incubated at 37°C for 120 min (Sun et al. 2022). The surviving cell number was checked by using a plate‐counting method by taking 100 μL samples to MRS Agar medium and incubating for 48 h at 37°C after 15 min of digestion in the homogenizer at high speed. The uncapsulated culture was evaluated as control group for same time intervals.

### 2.4 Development of Oral Spray Formulations

The probiotic oral spray formulations were prepared using purified water as the main solvent system. The excipients employed in the formulation included maltodextrin, gum arabic, glycerin, xylitol, ascorbic acid, sodium bicarbonate, sodium chloride, sodium benzoate, potassium sorbate, and sorbitol 70%. These auxiliary agents were incorporated at varying concentrations depending on the targeted functional properties of each formulation, such as viscosity, stability, osmolarity, and preservation. Initially, sodium hyaluronate (0.2%, w/v) was dispersed in purified water under continuous magnetic stirring to allow complete hydration and formation of a homogeneous mucoadhesive base. Licorice ( *Glycyrrhiza glabra* ) extract was subsequently incorporated into the hydrated polymer matrix at a concentration of 1% (w/v), ensuring uniform distribution. Humectants (glycerin and sorbitol 70%), sweeteners (xylitol), buffering agents (sodium bicarbonate and sodium chloride), antioxidants (ascorbic acid), and preservatives (sodium benzoate and potassium sorbate) were then added sequentially under controlled stirring conditions to obtain a clear and homogeneous solution. Following complete dissolution of all excipients, the previously prepared microencapsulated *Lactiplantibacillus pentosus* was incorporated into the formulation under gentle agitation to prevent structural damage to the microcapsules. The final probiotic concentration was adjusted to approximately 10^9^ CFU/mL using a densitometer according to the McFarland turbidity standard (McFarland 4). The standardized microencapsulated probiotic preparation was then uniformly dispersed within the formulation matrix to ensure a consistent probiotic concentration across all formulations. The finished formulations were aseptically filled into sterile spray containers and stored at 4°C until further biological activity, stability, and characterization analyses.

### 2.5 Biological Activity Analyses of the Formulations

#### 2.5.1 Antimicrobial Activity

Antimicrobial activity was evaluated using agar well diffusion and broth microdilution methods in accordance with Clinical and Laboratory Standards Institute (CLSI) guidelines. Antibacterial assays were performed following CLSI M02‐A11 and M07‐A9 standards, while antifungal assays were conducted according to CLSI M44‐A2 and M27‐A2 protocols with minor modifications. All assays were performed in triplicate. The antimicrobial activity of the formulations was tested against *Streptococcus mutans* ATCC 25175, *Streptococcus pyogenes* ATCC 19615, *Streptococcus sanguinis* ATCC 10556, *Staphylococcus aureus* ATCC 29213, *Pseudomonas aeruginosa* ATCC 27853, *Candida parapsilosis* ATCC 22019, and *Candida albicans* ATCC 90028. These microorganisms were selected to represent clinically relevant members of the oral microbiota as well as opportunistic pathogens associated with oral dysbiosis, dental plaque formation, biofilm development, and oral infections. *Streptococcus mutans* was included because of its major role in dental caries and cariogenic biofilm formation, whereas *Streptococcus sanguinis* was selected as an early colonizer of dental plaque and a representative commensal streptococcal species involved in oral biofilm ecology. *Streptococcus pyogenes* and *Staphylococcus aureus* were included as clinically important Gram‐positive opportunistic pathogens that may be associated with oral and oropharyngeal infections. *Pseudomonas aeruginosa* was selected as a Gram‐negative opportunistic pathogen with intrinsic antimicrobial resistance and biofilm‐forming ability, allowing evaluation of the formulation against a more resistant bacterial phenotype. In addition, *Candida albicans* and *Candida parapsilosis* were included to assess the potential antifungal activity of the formulations against yeasts that may become clinically relevant under conditions of oral microbial imbalance or immunosuppression. Therefore, the selected test panel enabled evaluation of the formulations against both representative oral microorganisms and clinically relevant opportunistic pathogens, in line with the aim of developing a pharmabiotic oral spray intended to support oral microbial balance.

##### 2.5.1.1 Agar Well Diffusion Assay

Fresh 24‐h cultures were adjusted to 0.5 McFarland turbidity using a densitometer. A total of 100 μL of each microbial suspension was spread onto Mueller–Hinton Agar (for bacteria) or Sabouraud Dextrose Agar (for yeasts). After surface drying, 6 mm wells were aseptically punched into the agar, and formulations were added into each well. Plates were incubated at 37°C for 24 h, and inhibition zones were measured in millimeters. Chloramphenicol (for bacteria), ketoconazole (for yeasts), and triclosan were used as reference antimicrobial agents (Karakaya et al. 2026). In addition, licorice ( *Glycyrrhiza glabra* ) extract alone was tested at the same concentration used in the formulations (1%, w/v) using the agar well diffusion method to evaluate its contribution to the overall antimicrobial activity of the complete oral spray formulations.

##### 2.5.1.2 Determination of Minimum Inhibitory Concentration (MIC)

The minimum inhibitory concentrations (MICs) of the developed formulations were determined using the broth microdilution method in accordance with CLSI standards (CLSI M07‐A9, M11‐A6 for bacteria; CLSI M27‐A2 for yeasts). Fresh 24‐h cultures of *Streptococcus mutans* , *Streptococcus pyogenes* , *Streptococcus sanguinis* , *Staphylococcus aureus* , *Pseudomonas aeruginosa* , *Candida parapsilosis* , and *Candida albicans* were used in the assays. Microbial suspensions were adjusted to 0.5 McFarland turbidity using a densitometer and further diluted in the appropriate growth medium to obtain a final inoculum concentration of approximately 5 × 10^5^ CFU/mL. Two‐fold serial dilutions of each formulation were prepared directly in sterile 96‐well microplates. Briefly, 100 μL of sterile broth medium (MHB for bacteria, RPMI 1640 for yeasts) was added to each well. Subsequently, 100 μL of the formulation was added to the first well and mixed thoroughly. Two‐fold serial dilutions were then performed across the plate by transferring 100 μL from one well to the next, ensuring equal dilution stepsThe formulations were two‐fold serially diluted to obtain concentrations ranging from 1 to 1/128 (1, 1/2, 1/4, 1/8, 1/16, 1/32, 1/64, and 1/128). After preparation of serial dilutions, 100 μL of standardized microbial inoculum was added to each well, resulting in a final volume of 200 μL per well. Microplates were incubated at 37°C for 24 h. Following incubation, 20 μL of 0.01% (w/v) resazurin solution was added to each well and plates were incubated for an additional 2 h at 37°C. Wells that showed no color change (blue/purple) were considered to indicate inhibition of microbial growth. MIC values were defined as the lowest concentration of the formulation that completely prevented visible growth. Positive growth controls (medium + inoculum), negative sterility controls (medium only), and reference antimicrobial agents (chloramphenicol for bacteria, ketoconazole for yeasts, and triclosan) were included to validate the assay (Alper Öztürk et al. 2021; A. Alper Öztürk and Aygül 2025; A. Alper Öztürk et al. 2019). Because the tested samples consisted of complete oral spray formulations rather than purified antimicrobial compounds, MIC values were expressed as formulation dilution factors (1–1/128) instead of μg/mL concentrations. This approach enables evaluation of the antimicrobial efficacy of the final formulation as administered and has been commonly applied in studies involving complex multi‐component formulations.

#### 2.5.2 Antibiofilm Activity

The antibiofilm activity of the developed formulations was evaluated using a modified Minimum Biofilm Eradication Concentration (MBEC) assay as described by (Cruz et al. 2018). The experiment was performed in triplicate.

Biofilm‐forming reference strains, including *Staphylococcus aureus* ATCC 29213 and *Streptococcus mutans* ATCC 25175 were used in the experiments. For the antibiofilm studies, *Streptococcus mutans* and *Staphylococcus aureus* were selected due to their well‐documented biofilm‐forming abilities and clinical relevance to oral health. *Streptococcus mutans* is considered one of the primary etiological agents of dental plaque formation and dental caries because of its capacity to adhere to tooth surfaces and produce extracellular polysaccharides that facilitate biofilm development. *Staphylococcus aureus* , although not a dominant member of the healthy oral microbiota, is an opportunistic pathogen that can colonize the oral cavity and form persistent biofilms associated with oral and systemic infections. Therefore, these microorganisms were selected to evaluate the potential of the developed oral spray formulation to inhibit biofilm formation by both cariogenic and opportunistic biofilm‐forming pathogens relevant to oral health. Microbial suspensions were prepared at 0.5 McFarland turbidity (approximately 10^8^ CFU/mL) and inoculated into appropriate growth media (Tryptic Soy Broth). Aliquots of 200 μL of each microbial suspension were transferred into the wells of sterile 96‐well microplates and incubated at 37°C for 48 h to allow biofilm formation. Following incubation, the planktonic phase was carefully removed, and wells were gently washed 2–3 times with sterile physiological saline solution to eliminate non‐adherent cells. Two‐fold serial dilutions of the formulations were prepared in fresh growth medium and 100 μL of each dilution was added to the corresponding wells containing pre‐formed biofilms. Plates were incubated for an additional 24 h at 37°C. After treatment, biofilm biomass was quantified by measuring optical density at 550 nm using a microplate spectrophotometer (Kaya et al. 2025).

The percentage of biofilm inhibition was calculated using the following Equation (3):

(3) $$\text{Inhibition}\%=\frac{OD_{\text{control}}-OD_{\text{sample}}}{OD_{\text{control}}}\times 100$$

All experiments were performed in triplicate, and results were expressed as mean values. The antibiofilm activity of the four different formulations against the tested pathogenic microorganisms will be evaluated based on the Minimum Biofilm Eradication Concentration ≥ 50% (MBEC_50_) criterion.

#### 2.5.3 Antioxidant Activity

The antioxidant capacity of the developed formulations was evaluated using ABTS, DPPH, and ORAC assays. Each analysis was performed in triplicate.

##### 2.5.3.1 ABTS Radical Scavenging Assay

ABTS radical scavenging activity was evaluated according to Re et al. (1999). The ABTS radical cation (ABTS^+^) was generated by mixing 7 mM ABTS solution with 2.5 mM potassium persulfate in equal volumes and incubating the mixture in the dark for 16 h at room temperature. The resulting ABTS^+^ solution was diluted with distilled water to obtain an absorbance of 0.70 ± 0.02 at 734 nm. For the assay, 990 μL of the standardized ABTS solution was mixed with 10 μL of each formulation. The reaction mixture was incubated at room temperature in the dark for 30 min, and absorbance was measured at 734 nm using a UV spectrophotometer. Ascorbic acid and gallic acid were used as reference antioxidants. Radical scavenging activity was calculated using the following Equation (4):

(4) $$\text{Inhibition}\%=\frac{A_{\text{control}}-A_{\text{sample}}}{A_{\text{control}}}\times 100$$

where $A_{\text{control}}$ = absorbance of control solution. $A_{\text{sample}}$ = absorbance of test sample.

##### 2.5.3.2 Free Radical Scavenging Assay (DPPH Test)

The DPPH radical scavenging activity was evaluated according to the method of Blois (1958). Serial two‐fold dilutions of the formulations were prepared in microplate wells. Subsequently, 100 μL of standardized DPPH solution and formulations were added to each well. The mixtures were incubated in the dark at room temperature for 60 min. Absorbance was then measured at 517 nm. Ascorbic acid and gallic acid were used as positive controls. The percentage of radical inhibition was calculated using the same equation described for the ABTS assay (Alper Öztürk et al. 2023; Solak et al. 2025).

##### 2.5.3.3 Oxygen Radical Absorbance Capacity (ORAC) Assay

The ORAC assay was performed following a modified method described by Huang et al. (2002). A standardized fluorescein solution was prepared and added to 96‐well microplates containing two‐fold serial dilutions of the formulations. Plates were incubated at 37°C in the dark for 30 min. Subsequently, 25 μL of 2,2′‐Azobis(2‐amidinopropane) dihydrochloride (AAPH) solution was added to initiate peroxyl radical formation. Fluorescence measurements were recorded at excitation/emission wavelengths of 485–520 nm every minute for 90 min using a microplate reader (Gen5 software). Trolox (12.5–50 μM) was used as the reference standard. ORAC values were calculated as percentage inhibition using the following equation (5):

(5) $$\text{ORAC}\%=\frac{I_{2}-I_{1}}{I_{0}-I_{1}}\times 100$$

where $I_{0}$ = initial fluorescein intensity; $I_{1}$ = fluorescein intensity in the absence of sample/standard; $I_{2}$ = fluorescein intensity in the presence of sample/standard.

### 2.6 Microbiological Contamination Analysis

Microbiological quality control was carried out according to ISO 21149 and ISO 16212 standards. At each stability interval, samples were spread‐plated onto Tryptic Soy Agar supplemented with Polysorbate 80/Lecithin for total aerobic microbial count and onto Sabouraud 4% Dextrose Agar for yeast and mold detection. Plates were incubated at 25°C and 37°C, and microbial growth was monitored. All analyses were performed in triplicate.

### 2.7 Determination of Probiotic Viable Count During Storage

Probiotic viability was monitored throughout the stability study at the designated time points. For viable count determination, formulations were homogenized for 15 min to facilitate the release of encapsulated bacterial cells. Serial dilutions were prepared using sterile peptone water, and aliquots were plated onto MRS agar. Plates were incubated at 37°C for 48 h, after which colonies were counted and results were expressed as CFU/mL. This analysis enabled evaluation of the stability of *Lactiplantibacillus pentosus* within the oral spray matrix under different storage conditions.

### 2.8 pH Measurement

The pH values of the formulations were determined using a calibrated digital pH meter at room temperature (Mettler ToledoTM S220 SevenCompact pH/lon Benchtop Meter). The instrument was calibrated using standard buffer solutions prior to analysis. Measurements were performed in triplicate for each sample, and the results were expressed as mean ± standard deviation (SD). To evaluate pH stability, the formulations were stored under refrigerated (4°C ± 2°C), room temperature (25°C ± 2°C/60% ± 5% RH), and accelerated (40°C ± 2°C/75% ± 5% RH) conditions for 3 months, and pH measurements were performed at the initial time and after 1, 2, and 3 months of storage (Çevikelli et al. 2024; Ekinci et al. 2025; Yenilmez et al. 2023).

### 2.9 Rheological Analysis

The rheological behavior of the formulations was evaluated using a rotational viscometer (Brookfield). Measurements were carried out at different shear rates, and the obtained data were fitted to the Casson model to determine plastic viscosity (PV), yield stress (YS), and coefficient of fit (COF). These parameters were used to characterize the flow properties of the oral spray formulations. For stability evaluation, the samples were stored at 4°C ± 2°C, 25°C ± 2°C/60% ± 5% RH, and 40°C ± 2°C/75% ± 5% RH for 3 months, and rheological measurements were repeated monthly (Ismailovi et al. 2024; A. Alper Öztürk et al. 2018; Öztürk and Güven 2019).

### 2.10 Organoleptic Evaluation

Organoleptic properties of the formulations were evaluated in terms of taste, color, odor, and appearance. Samples were visually inspected for any changes such as discoloration, phase separation, precipitation, or loss of homogeneity. Odor and taste were assessed based on their characteristic properties. For stability evaluation, the samples were stored at 4°C ± 2°C, 25°C ± 2°C/60% ± 5% RH, and 40°C ± 2°C/75% ± 5% RH for 3 months, and organoleptic characteristics were evaluated monthly (Öztürk et al. 2020; Xiao et al. 2021).

### 2.11 Statistical Analysis

All experiments and analytical measurements were performed in triplicate (*n* = 3), and the results were expressed as mean ± standard deviation (SD). Statistical analyses were conducted using GraphPad Prism version 8.0 (GraphPad Software, San Diego, CA, USA). Comparisons among multiple groups were performed using one‐way analysis of variance (one‐way ANOVA), followed by Tukey's multiple comparison post hoc test to identify statistically significant differences between individual groups. When comparisons involved only two groups, an unpaired Student's *t*‐test was applied, where appropriate. Differences were considered statistically significant at *p* < 0.05. Statistical significance levels are indicated as follows: *p < 0.05, **p < 0.01, ***p < 0.001, and ****p < 0.0001.

## 3 Results and Discussion

### 3.1 Encapsulation Efficiency and Viable Counts

High probiotic survival and encapsulation efficiency were achieved by freeze‐drying microencapsulation utilizing maltodextrin (MD) and gum arabic (GA) at a 1:1 ratio (total solids 30% w/v). Before encapsulation, the initial bacterial suspension had about 10^9^ CFU/mL. The viable count of *Lactiplantibacillus pentosus* after lyophilization and subsequent release of entrapped cells was found to be 8.17 ± 0.05 log CFU/mL (mean ± SD, *n* = 3), indicating that the freeze‐drying procedure only slightly reduced cell viability in comparison to the original inoculum (*p* > 0.05). A significant degree of probiotic encapsulation within the carbohydrate‐based polymeric matrix was indicated by the MD:GA (1:1) system's encapsulation efficiency (EE%) of 94.74% ± 1.12%. The reproducibility and robustness of the encapsulation process were confirmed by statistical analysis, which showed no significant variability across repeated measurements (*p* > 0.05).

### 3.2 Characterization of the Microcapsules

#### 3.2.1 Morphological and Particle Size Analysis

**FIGURE 2:**
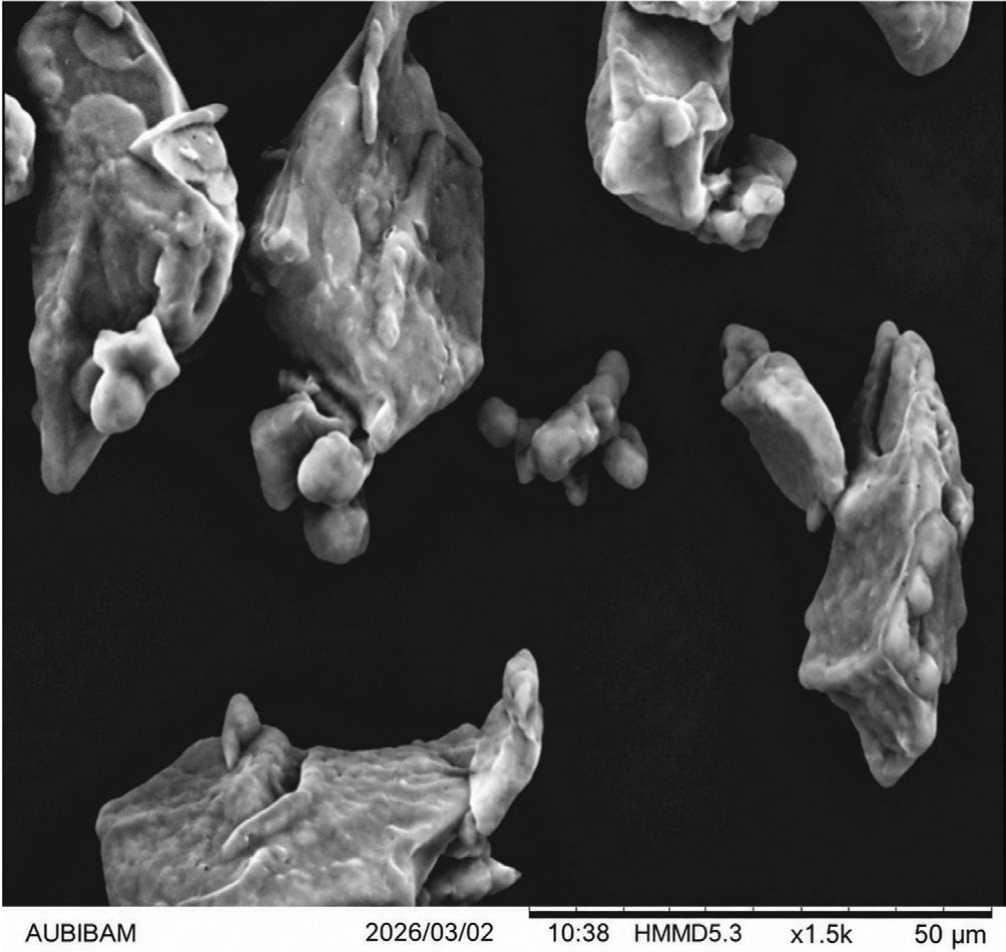
Scanning electron microscopy (SEM) micrograph (1500× magnification) of freeze‐dried *Lactiplantibacillus pentosus mic*rocapsules prepared using maltodextrin and gum arabic (1:1, w/w) as wall materials. Scale bar = 50 μm.

Scanning electron microscopy (SEM) was used to evaluate the surface morphology and particle characteristics of freeze‐dried *Lactiplantibacillus pentosus* microcapsules prepared using maltodextrin (MD) and gum arabic (GA) as encapsulating agents. The samples were examined under 500×, 1000×, and 1500× magnifications. Representative SEM micrographs obtained at 1500× magnification are presented in Figure 2.

The SEM micrographs revealed irregularly shaped microcapsules with dented and partially collapsed surface structures, which are characteristic of freeze‐dried probiotic microcapsules. The observed morphology is likely associated with water sublimation and matrix shrinkage occurring during the lyophilization process (Da Silva et al. 2019; Savedboworn et al. 2017). In addition, a certain degree of particle aggregation was observed, which is commonly reported for freeze‐dried probiotic powders. The mean particle diameter of the MD:GA (1:1) microcapsules was 36.6 ± 1.2 μm, which falls within the size range generally considered suitable for incorporation into food and pharmaceutical formulations (Lawless and Heymann 1999). The observed morphology is consistent with the functional roles of maltodextrin and gum arabic during freeze‐drying. Gum arabic contributes strong film‐forming and emulsifying properties, whereas maltodextrin promotes matrix vitrification and increases glass transition temperature, thereby enhancing structural stability during dehydration. As a result, the MD microcapsules formed coherent protective matrices surrounding the probiotic cells while maintaining a porous structure that may facilitate subsequent rehydration and release of viable microorganisms. Similar findings have been reported previously for probiotic microcapsules prepared using maltodextrin‐ and gum arabic‐based wall systems (Gullifa et al. 2023; Xiao et al. 2022). The dented surface morphology observed in the SEM images is typical of lyophilized probiotic microcapsules and results from the removal of ice crystals during primary drying. According to previous studies, freeze‐drying commonly produces porous and collapsed surface structures due to sublimation‐induced matrix contraction and the formation of internal voids during dehydration (Rockinger et al. 2021). From a functional perspective, particle size and morphology may influence moisture uptake, storage stability, rehydration behaviour, and probiotic release characteristics. Larger particles with coherent wall structures may provide improved protection during storage, while maintaining the ability to rapidly rehydrate upon contact with aqueous environments (Huang et al. 2023). The particle size obtained in the present study suggests that the MD wall system provided microcapsules with suitable structural characteristics for potential oral delivery applications (McNamee et al. 1998).

#### 3.2.2 Moisture Content and Hygroscopicity Analysis

The moisture content of lyophilized probiotic microcapsules is an important factor affecting the stability and viability of encapsulated microorganisms during storage. Freeze‐dried microcapsules may exhibit a tendency to agglomerate when residual moisture levels increase, while their porous structure generally promotes rapid reconstitution upon contact with water (Barbosa et al. 2015). Previous studies have reported that moisture contents between 4% and 5% provide favorable storage conditions for encapsulated probiotic cultures (Ranadheera et al. 2015). Hygroscopicity, defined as the ability of powders to absorb moisture from the surrounding environment, is also closely associated with probiotic stability and survival during storage, particularly under conditions of elevated relative humidity (Bhandari et al. 2024). In the present study, lyophilized *Lactiplantibacillus pentosus* microcapsules prepared using maltodextrin and gum arabic (MD, 1:1) exhibited a moisture content of 5.70% and a hygroscopicity value of 13.55%. The microcapsules demonstrated rapid reconstitution in water, which may be attributed to the porous structure generated during the lyophilization process. The observed moisture content was slightly above the optimal range reported in the literature, suggesting that appropriate packaging and moisture‐control strategies may be beneficial to maintain long‐term stability. Residual moisture is considered one of the major factors influencing probiotic shelf life, as elevated moisture levels may increase particle stickiness, agglomeration, and molecular mobility within the matrix (Ranadheera et al. 2015). The moisture content obtained for the MD microcapsules indicates generally acceptable storage stability while highlighting the importance of controlling environmental humidity during storage. Similarly, the hygroscopicity value obtained in this study suggests a moderate tendency for moisture uptake under humid conditions. Therefore, storage under low relative humidity conditions, together with the use of moisture‐barrier packaging materials and desiccant systems, may help minimize moisture absorption and preserve probiotic viability (Kurtmann et al. 2009). The results indicate that the MD wall system produced microcapsules with acceptable moisture content and hygroscopicity characteristics for probiotic delivery applications. Nevertheless, further optimization of drying and packaging conditions may contribute to improved storage stability and reduced moisture‐related deterioration during prolonged storage (Kurtmann et al. 2009).

#### 3.2.3 Viability of Microencapsulated Probiotics During the In Vitro Digestion Process

**FIGURE 3:**
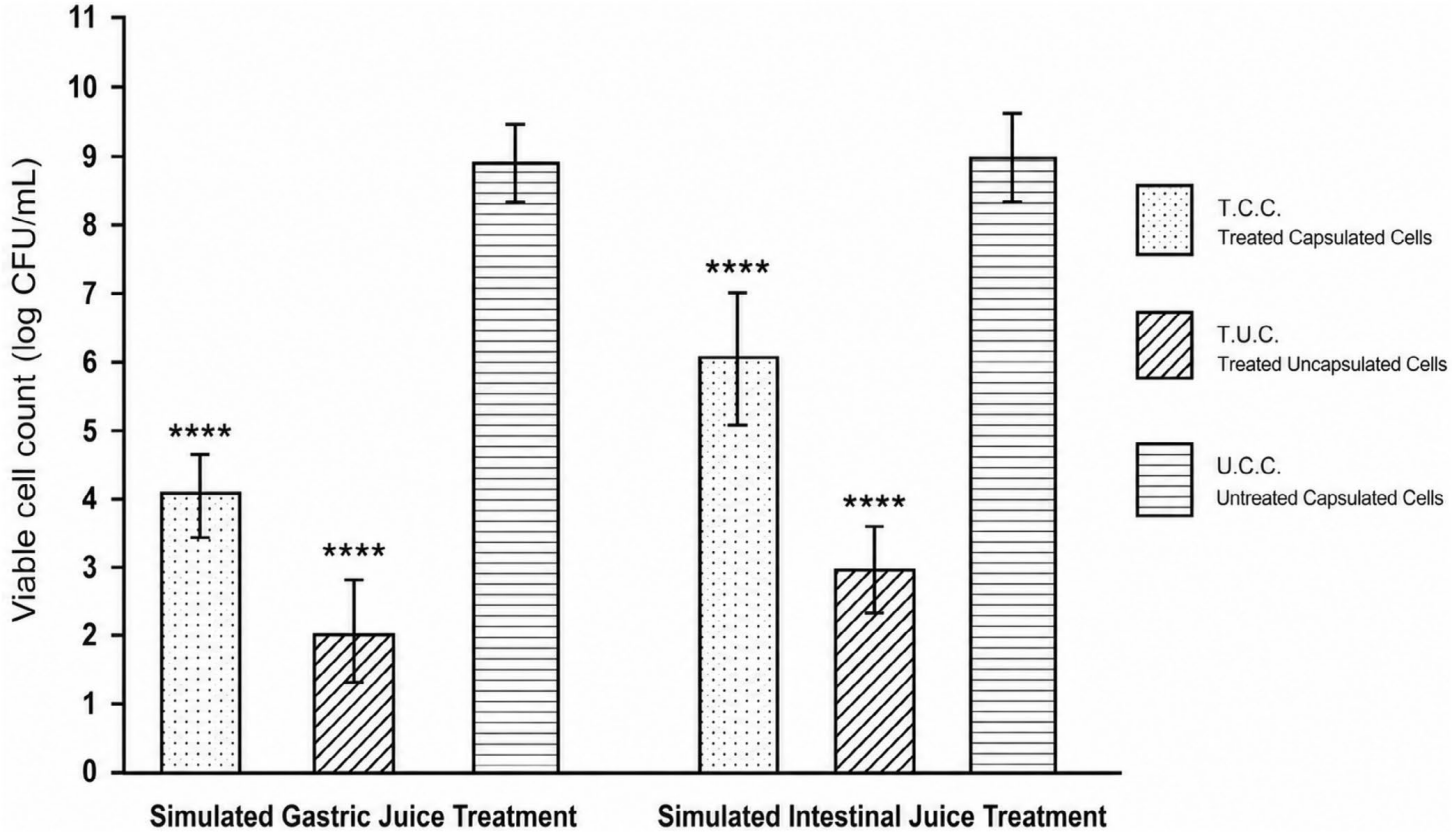
Survival of *Lactiplantibacillus pentosus mic*rocapsules prepared with maltodextrin–gum arabic (MD:GA, 1:1) under simulated gastrointestinal conditions (****p < 0.0001). Data are presented as mean ± standard deviation (*n* = 3).

Under static in vitro digestion conditions, microencapsulation significantly improved the survival of *Lactiplantibacillus pentosus* compared with non‐encapsulated cells (*p* < 0.001). The untreated encapsulated control maintained viability close to 9 log CFU/mL throughout the experiment, indicating that the encapsulation system effectively preserved probiotic viability in the absence of digestive stress. Following exposure to simulated gastrointestinal conditions for 120 min, the MD (maltodextrin–gum arabic, 1:1) microcapsules retained approximately 4 log CFU/mL viable cells in simulated gastric juice and approximately 6 log CFU/mL viable cells in simulated intestinal juice. In contrast, the viability of treated unencapsulated cells decreased to approximately 2 log CFU/mL and 3 log CFU/mL under simulated gastric and intestinal conditions, respectively. The mean results obtained for simulated gastric and intestinal digestion are presented in Figure 3.

Efficient delivery of viable probiotics to the gastrointestinal tract is essential for achieving their intended health benefits; therefore, the selection of appropriate wall materials plays a critical role in protecting probiotic cells against harsh environmental conditions encountered during digestion (Chandra Baral et al. 2021). The markedly higher viability of encapsulated *L. pentosus* compared with unencapsulated cells demonstrates the protective capacity of the MD:GA wall system against gastrointestinal stress. The protective effect of the microcapsules may be attributed to the complementary functional properties of maltodextrin and gum arabic. Gum arabic contributes a highly soluble film‐forming arabinogalactan–protein network that enhances structural integrity and encapsulation efficiency, whereas maltodextrin promotes matrix vitrification and stabilization of cellular structures during dehydration and rehydration processes (Kamwa et al. 2024; Zheng et al. 2015). Together, these wall materials form a protective matrix capable of reducing the detrimental effects of acidic pH, digestive enzymes, and bile salts encountered during gastrointestinal transit. Similar protective effects of maltodextrin–gum arabic systems have previously been reported for probiotic microencapsulation applications (Misra et al. 2022). Although viability losses were observed under simulated digestion conditions, encapsulated cells consistently exhibited significantly higher survival than unencapsulated cells. Interestingly, probiotic survival was greater under simulated intestinal conditions than under simulated gastric conditions, suggesting that exposure to the acidic gastric environment represented the primary factor responsible for viability loss. The approximately 5‐log reduction observed under gastric conditions compared with the untreated control indicates that low pH remains a substantial challenge for probiotic survival despite encapsulation. Nevertheless, the approximately 3‐log reduction observed under intestinal conditions demonstrates that the MD:GA matrix was able to maintain a considerable proportion of viable cells throughout the digestion process. The enhanced survival of encapsulated cells may also be associated with hydrogen‐bond interactions between the wall materials and cellular structures, which help stabilize bacterial membranes and proteins during exposure to stressful environmental conditions (Liao et al. 2017; Santivarangkna et al. 2008). Overall, the results indicate that microencapsulation using maltodextrin and gum arabic significantly improved the gastrointestinal tolerance of *L. pentosus* and may represent an effective strategy for enhancing probiotic survival during oral administration. These findings support the potential application of MD:GA microcapsules in pharmaceutical and nutraceutical formulations where maintenance of probiotic viability during gastrointestinal transit is essential for functionality and therapeutic efficacy.

### 3.3 Development of Oral Spray Formulations

To maximize physicochemical stability, bacterial viability, and biological functioning, four distinct probiotic oral spray formulations (F1–F4) were created. In addition to sodium hyaluronate (0.2%, w/v) as a mucoadhesive polymer and licorice extract (1%, w/v) as a phytochemical bioactive agent, all formulations included microencapsulated *Lactiplantibacillus pentosus* at a target concentration of around 10^9^ CFU/mL. To assess their effects on viscosity, clarity, organoleptic qualities, and probiotic compatibility, the formulations varied in the relative amounts of humectants, buffering agents, and stabilizing excipients (Table 1).

**TABLE 1:** Quantitative composition of F1–F4 probiotic oral spray formulations (% w/v).

Component	F1	F2	F3	F4
Microencapsulated *L. pentosus*	10^9^ CFU/mL	10^9^ CFU/mL	10^9^ CFU/mL	10^9^ CFU/mL
Sodium hyaluronate	0.20	0.20	0.20	0.20
Licorice extract	1.00	1.00	1.00	1.00
Glycerin	5.00	7.50	10.00	12.50
Sorbitol 70%	3.00	5.00	7.00	8.00
Xylitol	2.00	2.00	2.50	3.00
Ascorbic acid	0.10	0.15	0.20	0.25
Sodium bicarbonate	0.20	0.20	0.20	0.20
Sodium chloride	0.40	0.45	0.50	0.55
Sodium benzoate	0.10	0.10	0.10	0.10
Potassium sorbate	0.10	0.10	0.10	0.10
Purified water	100 mL qs	100 mL qs	100 mL qs	100 mL qs

Four formulations (F1–F4) were developed to investigate the influence of selected excipients on the physicochemical characteristics, stability, and biological performance of the oral spray. The concentrations of glycerin, sorbitol solution (70%, w/w), xylitol, ascorbic acid, and sodium bicarbonate were varied based on preliminary formulation trials and literature reports regarding their use in oral pharmaceutical and oral care products. These excipients were selected because they may influence formulation viscosity, organoleptic properties, pH stability, and overall product performance. In contrast, the concentration of microencapsulated *Lactiplantibacillus pentosus* was intentionally maintained at approximately 10^9^ CFU/mL in all formulations to ensure delivery of a standardized and biologically relevant probiotic dose. Probiotic products are generally recommended to contain at least 10^6^–10^8^ CFU per serving at the time of consumption, while concentrations around 10^9^ CFU/mL are frequently employed in formulation studies to ensure adequate viability and functionality throughout storage and administration. Therefore, a constant probiotic concentration was selected to allow the effects of formulation excipients on physicochemical stability and biological performance to be evaluated independently. The formulation exhibiting the most favorable combination of physicochemical stability, probiotic viability, organoleptic properties, rheological behavior, and biological activity was considered the optimum formulation. A formal design‐of‐experiments (DoE) approach was not employed in the present study; however, the selected formulation compositions were established through preliminary optimization studies and formulation development considerations.

### 3.4 Antimicrobial Activity of the Oral Spray Formulations

**FIGURE 4:**
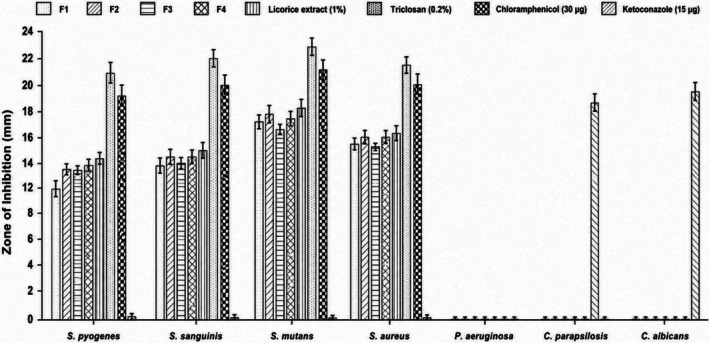
Antimicrobial zone inhibition values of (*p* < 0.0001) of formulations. Data are expressed as mean ± SD (*n* = 3).

The antimicrobial activity of the developed probiotic oral spray formulations was evaluated using agar well diffusion and broth microdilution assays against representative oral bacterial and fungal pathogens. The inhibition zone diameters and minimum inhibitory concentration (MIC) values obtained for the formulations and reference antimicrobial agents are presented in Figure 4 and Tables 2 and 3.

**TABLE 2:** Antimicrobial inhibition diameters (mm) of the formulations.

Samples	*S. pyogenes*	*S. sanguinis*	*S. mutans*	*S. aureus*	*P. aeruginosa*	*C. parapsilosis*	*C. albicans*
F1	7.4 ± 0.2	7.8 ± 0.4	12.1 ± 0.3	10.5 ± 0.3	—	—	—
F2	7.4 ± 0.2	7.8 ± 0.4	12.1 ± 0.3	10.5 ± 0.3	—	—	—
F3	7.4 ± 0.2	7.8 ± 0.4	12.1 ± 0.3	10.5 ± 0.3	—	—	—
F4	7.4 ± 0.2	7.8 ± 0.4	12.1 ± 0.3	10.5 ± 0.3	—	—	—
Licorice extract	4.5 ± 0.5	3.0 ± 0.2	4.0 ± 0.6	4.0 ± 0.5	—	—	—
Triclosan	19.3 ± 0.3	18.4 ± 0.2	18.4 ± 0.5	16.4 ± 0.5	16.5 ± 0.2	—	—
Chloramphenicol	22.4 ± 0.3	20.3 ± 0.2	20.4 ± 0.3	22.4 ± 0.3	12.6 ± 0.2	—	—
Ketoconazole	—	—	—	—	—	23.4 ± 0.3	28.4 ± 0.4

**TABLE 3:** MIC values of the formulations.

Samples	*S. pyogenes*	*S. sanguinis*	*S. mutans*	*S. aureus*	*P. aeruginosa*	*C. parapsilosis*	*C. albicans*
F1	1/8	1/8	1/16	1/16	—	—	—
F2	1/8	1/8	1/16	1/16	—	—	—
F3	1/8	1/8	1/16	1/16	—	—	—
F4	1/8	1/8	1/16	1/16	—	—	—
Triclosan (μg/mL)	0.001	0.0005	0.001	0.0005	0.001	0.016	0.032
Chloramphenicol (μg/mL)	0.016	0.016	0.008	0.004	0.008	—	—
Ketoconazole (μg/mL)	—	—	—	—	—	0.0005	0.000125

In the agar well diffusion assay, all formulations (F1–F4) exhibited measurable antibacterial activity against Gram‐positive oral bacteria, including *Streptococcus mutans* , *Streptococcus pyogenes* , *Streptococcus sanguinis* , and *Staphylococcus aureus* (Table 2). The inhibition zone diameters were highly consistent across all formulations. The largest inhibition zones were observed against *Streptococcus mutans* (12.1 ± 0.3 mm), followed by *Staphylococcus aureus* (10.5 ± 0.3 mm). Moderate inhibition zones were observed for *Streptococcus sanguinis* (7.8 ± 0.4 mm) and *Streptococcus pyogenes* (7.4 ± 0.2 mm). No inhibition zones were detected against the Gram‐negative bacterium *Pseudomonas aeruginosa* or the yeast strains *Candida parapsilosis* and *Candida albicans* . The similarity of inhibition zones among the four formulations indicates that variations in excipient composition did not significantly influence antimicrobial activity. Statistical analysis confirmed that inhibition zones of the formulations were significantly smaller than those produced by the reference antimicrobial agents (*p* < 0.0001), indicating a lower but measurable antibacterial effect compared with conventional antimicrobial compounds. Among the tested microorganisms, *Streptococcus mutans* showed the highest susceptibility to the formulations, suggesting that the developed probiotic oral spray may particularly target cariogenic bacteria involved in dental plaque formation and caries development. The absence of inhibitory activity against *Pseudomonas aeruginosa* can be attributed to the intrinsic resistance mechanisms associated with Gram‐negative bacteria. The outer membrane structure of these bacteria restricts the penetration of many antimicrobial compounds and reduces susceptibility to plant‐derived or probiotic‐derived metabolites. Similarly, no inhibition zones were observed for *Candida* species, suggesting that the concentration of active components within the formulations was insufficient to produce antifungal inhibition under agar diffusion conditions. To further investigate the contribution of licorice extract to the antimicrobial activity of the formulations, licorice extract alone (1%, w/v) was evaluated using the agar well diffusion assay. The extract exhibited antibacterial activity against the tested Gram‐positive oral pathogens, including *Streptococcus mutans* , *Staphylococcus aureus* , *Streptococcus sanguinis* , and *Streptococcus pyogenes* . No inhibition zones were observed against *Pseudomonas aeruginosa* , *Candida albicans* , or *Candida parapsilosis* . The inhibition pattern obtained for licorice extract was comparable to that observed for the complete formulations, suggesting that licorice‐derived bioactive compounds contributed substantially to the antibacterial activity of the developed oral spray formulations. The broth microdilution assay results were consistent with the agar diffusion findings. All formulations demonstrated identical MIC values against the tested Gram‐positive bacteria (Table 3). The MIC values were determined as a 1/16 dilution for *Streptococcus mutans* and *Staphylococcus aureus* , and a 1/8 dilution for *Streptococcus pyogenes* and *Streptococcus sanguinis* . No inhibitory activity was detected against *Pseudomonas aeruginosa* or the tested *Candida* species within the concentration range evaluated. The identical MIC values obtained for F1–F4 further confirm that the antimicrobial activity of the formulations is primarily related to the biological components of the formulation rather than differences in excipient ratios. It should also be noted that several formulation excipients, including glycerin, sorbitol, xylitol, sodium bicarbonate, and sodium chloride, were primarily incorporated to provide physicochemical stability, humectancy, buffering capacity, and sensory acceptability rather than direct antimicrobial effects. The similar antimicrobial responses observed among formulations containing different concentrations of these excipients suggest that they contributed minimally to the antibacterial activity under the experimental conditions employed. Consequently, the observed antimicrobial effect is more likely associated with the biologically active ingredients, namely licorice extract and the probiotic component, rather than the auxiliary formulation excipients. In addition, the MIC values reported in the present study represent dilution factors of the complete oral spray formulations rather than concentrations of individual active compounds. Since the formulations contain multiple ingredients, including probiotic microcapsules, licorice extract, and pharmaceutical excipients, dilution‐based MIC determination was considered more appropriate for evaluating the antimicrobial performance of the final product as a whole. Therefore, the MIC results should be interpreted as indicators of the antimicrobial efficacy of the complete formulation rather than the potency of any individual component. When compared with the reference antimicrobial agents, the formulations showed lower antimicrobial potency. Triclosan demonstrated inhibition zones ranging between approximately 16.5 and 19.3 mm, while chloramphenicol produced inhibition zones up to approximately 22 mm against *Staphylococcus aureus* . Similarly, MIC values of triclosan and chloramphenicol were substantially lower than those of the formulations, indicating stronger antimicrobial activity. Ketoconazole exhibited strong antifungal activity against *Candida* species, producing inhibition zones exceeding 23 mm and very low MIC values. Although the developed formulations were less potent than conventional antimicrobial agents, the observed antibacterial activity is noteworthy because the formulations are designed as probiotic‐based oral care systems rather than antibiotic preparations. The antimicrobial effect observed in the present study may result from the combined activity of several formulation components. Licorice extract is known to contain bioactive compounds such as glycyrrhizin, flavonoids, and isoflavonoids that exhibit antibacterial activity against oral pathogens (Nassiri Asl and Hosseinzadeh 2008). In addition, probiotic lactic acid bacteria can produce antimicrobial metabolites including organic acids, bacteriocins, hydrogen peroxide, and other inhibitory compounds that suppress the growth of pathogenic microorganisms (Salminen et al. 2021). The additional evaluation of licorice extract alone demonstrated that the extract possesses intrinsic antibacterial activity against Gram‐positive oral pathogens. Licorice ( *Glycyrrhiza glabra* ) contains several bioactive constituents, including glycyrrhizin, glabridin, flavonoids, and isoflavonoids, which have been reported to inhibit the growth of cariogenic and opportunistic oral bacteria. The similarity between the inhibition patterns obtained for licorice extract and the complete formulations suggests that licorice extract represents a major contributor to the direct antibacterial activity observed in this study.

The absence of significant differences among formulations F1–F4 may be explained by the fact that both the concentration of licorice extract (1%, w/v) and the concentration of microencapsulated *L. pentosus* were maintained constant across all formulations. Therefore, variations in glycerin, sorbitol, xylitol, ascorbic acid, and sodium bicarbonate concentrations primarily affected physicochemical characteristics rather than antimicrobial performance. The inhibition pattern observed in this study is consistent with previous reports describing the antibacterial activity of probiotic or plant‐derived oral formulations. Previous studies have reported inhibition zones of approximately 10–15 mm against *Streptococcus mutans* for probiotic‐based or phytochemical‐based oral products, which is comparable to the inhibition zones observed in the present study. Similarly, earlier studies have reported limited activity of plant‐derived antimicrobials against *Pseudomonas aeruginosa* due to the protective role of the Gram‐negative outer membrane. The results indicate that the developed probiotic oral spray formulations possess moderate antibacterial activity primarily against Gram‐positive oral pathogens, particularly *Streptococcus mutans* . This selective antibacterial activity suggests that the formulations may contribute to the control of cariogenic bacteria while maintaining the ecological balance of the oral microbiota.

### 3.5 Antibiofilm Activity of the Oral Spray Formulations

**FIGURE 5:**
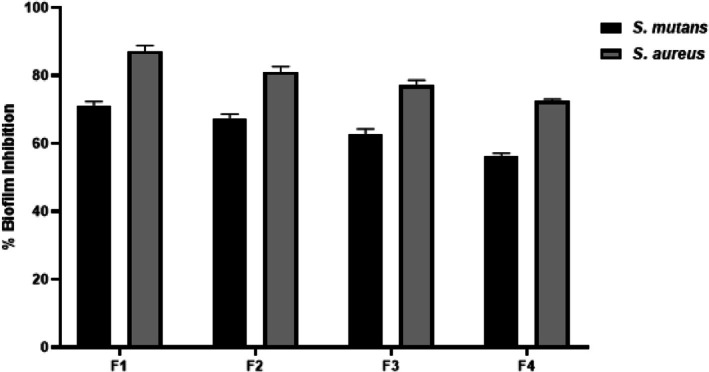
The % biofilm inhibition values (*p* < 0.0001) of formulations. Data are expressed as mean ± SD (*n* = 3).

The antibiofilm activity of the developed oral spray formulations was evaluated against *Streptococcus mutans* and *Staphylococcus aureus* , two clinically relevant biofilm‐forming oral pathogens. The results were assessed based on the Minimum Biofilm Eradication Concentration ≥ 50% (MBEC_50_) criterion. The percentage of biofilm inhibition obtained for all formulations is presented in Figure 5. All four formulations demonstrated considerable antibiofilm activity against both tested microorganisms, with inhibition values exceeding the MBEC_50_ threshold. For *S. mutans* , the percentage of biofilm inhibition ranged from 56.17% ± 0.91% to 70.99% ± 1.32%, whereas inhibition against *S. aureus* ranged from 72.42% ± 0.56% to 87.03% ± 1.76%. These findings indicate that all formulations were capable of significantly reducing biofilm formation by the tested pathogens. Among the tested formulations, F1 exhibited the highest antibiofilm activity against both microorganisms, producing inhibition values of 70.99% ± 1.32% for *S. mutans* and 87.03% ± 1.76% for *S. aureus* . In contrast, F4 showed comparatively lower inhibition values (56.17% ± 0.91% for *S. mutans* and 72.42% ± 0.56% for *S. aureus* ). The observed trend suggests that antibiofilm activity slightly decreased from F1 to F4. Despite these variations, all formulations maintained inhibition levels well above the MBEC_50_ threshold, confirming their strong antibiofilm potential. A comparison between the two microorganisms revealed that *S. aureus* biofilms were more susceptible to the formulations than *S. mutans* . This difference may be related to variations in biofilm architecture and extracellular polymeric substance composition between the two bacterial species. Biofilms formed by *S. mutans* are known to contain dense glucan‐rich matrices that contribute to increased structural stability and resistance to antimicrobial agents. Previous studies have reported antibiofilm inhibition rates ranging from approximately 40%–70% for plant‐derived oral formulations targeting *S. mutans* biofilms (Karygianni et al. 2016). In the present study, inhibition values reached approximately 71% for *S. mutans* and up to 87% for *S. aureus* , indicating that the developed probiotic oral spray formulations exhibit relatively strong antibiofilm activity compared with previously reported plant‐based oral formulations. Overall, the results demonstrate that the developed oral spray formulations possess significant antibiofilm activity against important oral pathogens. The ability of the formulations to inhibit biofilm formation above the MBEC_50_ threshold suggests their potential application in oral healthcare products aimed at preventing dental plaque formation and biofilm‐associated oral infections. These antibiofilm results were in agreement with the antimicrobial assays, which also indicated selective inhibitory activity of the formulations against Gram‐positive pathogens such as *Streptococcus mutans* and *Staphylococcus aureus* . The current study focused primarily on formulation development and in vitro characterization. Therefore, the broader effects of the formulation on the complex oral microbiome ecosystem remain to be elucidated through in vivo and clinical investigations.

### 3.6 Antioxidant Activity of the Oral Spray Formulations

**FIGURE 6:**
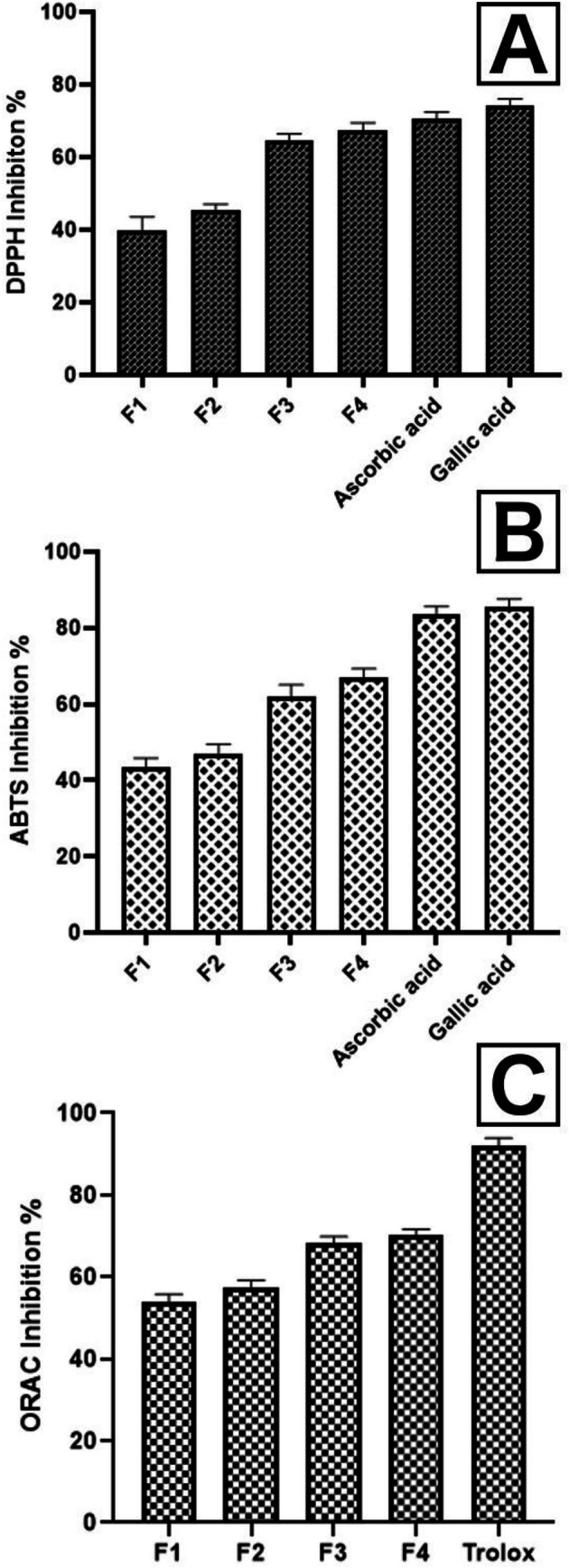
Antioxidant activities of the developed oral spray formulations. (A) DPPH radical scavenging activity, (B) ABTS radical scavenging activity, and (C) ORAC assay results of formulations F1–F4. Data are expressed as mean ± SD (*n* = 3).

The antioxidant activity of the developed probiotic oral spray formulations was evaluated using three complementary assays: DPPH radical scavenging activity, ABTS radical cation decolorization assay, and oxygen radical absorbance capacity (ORAC). These assays reflect different mechanisms of antioxidant action, including hydrogen atom transfer, electron transfer, and inhibition of peroxyl radical–mediated oxidation. Therefore, the combined interpretation of these assays provides a comprehensive understanding of the antioxidant potential of the formulations. In the DPPH assay, the formulations exhibited moderate radical scavenging activity, with inhibition values ranging from 39.63% ± 3.93% to 67.44% ± 2.06%. The results indicated that all formulations were capable of scavenging free radicals to varying degrees. While the inhibition values were lower than those observed for the standard antioxidants ascorbic acid (70.45% ± 2.07%) and gallic acid (74.21% ± 1.82%), the formulations demonstrated a measurable antioxidant effect. These findings suggest that the formulation matrix itself contributes to radical scavenging activity Figure 6a. A similar pattern was observed in the ABTS radical scavenging assay. The inhibition percentages ranged between 43.48% ± 2.36% and 67.03% ± 2.32%. The slightly higher inhibition values obtained in the ABTS assay compared with the DPPH assay may be related to the higher solubility of the ABTS radical in aqueous environments. Since the developed oral sprays are water‐based formulations, the ABTS assay may better reflect the antioxidant behavior of the formulations under physiological conditions Figure 6b. The ORAC assay further confirmed the antioxidant capacity of the formulations. ORAC inhibition values ranged from 53.75% ± 2.05% to 70.30% ± 1.31%, while the reference antioxidant Trolox exhibited an inhibition value of 92.08% ± 1.88%. The results obtained from the ORAC assay were generally higher than those observed in the DPPH assay, which is consistent with the fact that ORAC measures antioxidant activity against peroxyl radicals, one of the most biologically relevant reactive oxygen species Figure 6c. When the three antioxidant assays are evaluated together, a consistent antioxidant response can be observed across all formulations. Although some variability was detected among the formulations, the results of the DPPH, ABTS, and ORAC assays collectively indicate that the developed oral spray systems possess a moderate antioxidant capacity. The consistency between the assays suggests that the antioxidant activity observed is related to the formulation composition rather than methodological differences. The antioxidant properties of the formulations may be attributed to several components present in the formulation matrix. Ascorbic acid is a well‐known antioxidant that neutralizes reactive oxygen species through hydrogen atom donation and electron transfer mechanisms. In addition, licorice ( *Glycyrrhiza glabra* ) extract is rich in flavonoids and phenolic compounds that exhibit strong antioxidant properties. These compounds can scavenge free radicals and inhibit oxidative processes. The antioxidant activity observed in the developed formulations may be partially attributed to the presence of licorice ( *Glycyrrhiza glabra* ) extract, which is well known for its rich content of phenolic compounds, flavonoids, chalcones, and isoflavonoids. Several studies have demonstrated that licorice extracts possess substantial antioxidant activity in DPPH, ABTS, and other radical scavenging assays (Nassiri Asl and Hosseinzadeh 2008; Pastorino et al. 2018; Tohma et al. 2017). For example, ethanolic and hydroalcoholic extracts of *G. glabra* have been reported to exhibit strong DPPH radical scavenging activities, frequently exceeding 70%–90% inhibition at higher extract concentrations, while comparable antioxidant capacities have also been observed using ABTS‐based methods (Tohma et al. 2017). Furthermore, licorice extracts have demonstrated considerable oxygen radical absorbance capacity and reducing power, indicating an ability to neutralize peroxyl radicals and other reactive oxygen species (Pastorino et al. 2018). These antioxidant properties have largely been attributed to bioactive constituents such as glabridin, liquiritigenin, isoliquiritigenin, and glycyrrhizin, which are capable of donating hydrogen atoms or electrons to stabilize free radicals and interrupt oxidative chain reactions (Nassiri Asl and Hosseinzadeh 2008; Pastorino et al. 2018).

However, it should be emphasized that licorice extract was incorporated at a fixed concentration (1%, w/v) in all formulations. Therefore, the differences observed among formulations cannot be directly attributed to variations in licorice concentration. Instead, formulation‐dependent factors, including the concentrations of ascorbic acid, glycerin, sorbitol, xylitol, and sodium bicarbonate, may have influenced the stability, solubility, and accessibility of antioxidant compounds within the formulation matrix. Consequently, the present study demonstrates the antioxidant potential of the complete formulation rather than the dose‐dependent antioxidant effect of licorice extract alone. Future studies evaluating different concentrations of licorice extract and individual formulation components would provide a clearer understanding of their relative contributions and potential synergistic interactions. Previous studies have demonstrated that extracts of *Glycyrrhiza glabra* possess significant radical scavenging activity in both DPPH and ABTS assays due to their phenolic constituents (Nassiri Asl and Hosseinzadeh 2008). Furthermore, probiotic lactic acid bacteria, including species within the genus *Lactiplantibacillus*, have been reported to contribute to antioxidant activity through the production of bioactive metabolites such as exopolysaccharides, peptides, and organic acids. Therefore, the observed antioxidant activity in the present formulations may result from the combined effects of plant‐derived compounds, antioxidant excipients, and probiotic‐derived metabolites. When compared with previously reported plant‐based oral formulations, the inhibition values observed in this study fall within the range commonly reported for natural antioxidant systems. For example, plant extract–containing oral health formulations have been reported to exhibit DPPH and ABTS inhibition values generally ranging between 40% and 70%, depending on extract composition and concentration (Karygianni et al. 2016). Overall, the results obtained from the DPPH, ABTS, and ORAC assays indicate that the developed probiotic oral spray formulations possess a consistent and moderate antioxidant capacity. The antioxidant activity appears to originate from the combined presence of antioxidant excipients, licorice extract, and probiotic‐derived bioactive compounds. The results obtained from the DPPH, ABTS, and ORAC assays indicate that the developed probiotic oral spray formulations possess a consistent and moderate antioxidant capacity. The antioxidant activity appears to originate from the combined presence of antioxidant excipients, licorice extract, and probiotic‐derived bioactive compounds. However, it should be noted that the DPPH, ABTS, and ORAC assays are chemical antioxidant assays that evaluate radical scavenging capacity and antioxidant potential under in vitro conditions. Although these methods provide valuable preliminary information regarding the antioxidant characteristics of the formulations, they do not directly demonstrate biological antioxidant activity in oral tissues or cellular systems. Therefore, the results should be interpreted as evidence of antioxidant potential rather than confirmed biological efficacy. Future studies employing cell‐based oxidative stress models, intracellular reactive oxygen species (ROS) measurements, or oral epithelial cell systems are warranted to validate the biological relevance of the antioxidant activity observed in the present study.

### 3.7 Microbiological Contamination Analysis of the Formulations

Microbiological contamination analyses were performed concurrently with the stability studies in order to evaluate the microbiological safety and storage stability of the developed probiotic oral spray formulations. According to the analysis results, no microbial contamination was detected in any of the four formulations during the accelerated stability testing period. The total aerobic mesophilic bacteria count and yeast–mold count were determined to be < 10 cfu/mL in all formulations. These values are considerably lower than the acceptable microbial limits established by international cosmetic and pharmaceutical microbiological standards. According to ISO 21149 and ISO 16212 guidelines, the total aerobic microbial count (TAMC) and total yeast and mold count (TYMC) in oral or cosmetic formulations should generally remain below 10^2^ cfu/mL to ensure microbiological safety (ISO 16212:2017—Cosmetics—Microbiology—Enumeration of Yeast and Mould, n.d.; ISO 21149:2017—Cosmetics—Microbiology—Enumeration and Detection of Aerobic Mesophilic Bacteria, n.d.). Therefore, the values obtained in this study indicate that the developed formulations comply with internationally accepted microbiological quality requirements. The absence of detectable microbial growth during incubation suggests that the formulations remained microbiologically stable under the tested storage conditions. The microbiological stability of the formulations may be attributed to the combined effect of several formulation components. In particular, sodium benzoate and potassium sorbate are widely used preservative agents in oral and cosmetic formulations due to their ability to inhibit the growth of bacteria, yeasts, and molds. Sodium benzoate is known to exert antimicrobial activity by disrupting microbial cell membrane integrity and interfering with intracellular pH regulation, especially under slightly acidic conditions (Chipley 2020). Similarly, potassium sorbate is an effective preservative against fungi and yeast species and has been extensively used in food, pharmaceutical, and cosmetic formulations because of its broad antimicrobial spectrum and safety profile (Davidson et al. 2014). The combined use of these preservatives may enhance microbial stability through synergistic antimicrobial effects. In addition to the preservative system, other formulation components may also contribute to microbiological stability. Ascorbic acid present in the formulations can create mildly acidic conditions that are unfavorable for the growth of many pathogenic microorganisms. Acidic environments are known to suppress microbial proliferation by disrupting cellular metabolic processes and enzyme activity (Ray and Bhunia 2013). Furthermore, the inclusion of licorice ( *Glycyrrhiza glabra* ) extract in the formulations may provide additional antimicrobial support. Licorice has been reported to contain various bioactive compounds, including flavonoids, glycyrrhizin, and phenolic constituents, which exhibit antimicrobial activity against oral pathogens. Previous studies have demonstrated that licorice‐derived compounds can inhibit the growth of *Streptococcus mutans* and other oral bacteria involved in dental plaque formation (Karygianni et al. 2016). The microbiological safety of probiotic formulations is particularly important because contamination can compromise both product stability and probiotic viability. According to the World Health Organization and FAO guidelines for probiotic products, maintaining microbiological purity during storage is essential to ensure both product safety and functional efficacy. Overall, the microbiological analysis results indicate that the developed oral spray formulations maintained excellent microbiological quality throughout the stability study. The absence of detectable contamination suggests that the formulation composition, preservative system, and manufacturing conditions were effective in preventing microbial growth. These findings support the suitability of the formulations for safe oral use and long‐term storage.

### 3.8 Probiotic Viability During Stability Studies

The viability of *Lactiplantibacillus pentosus* in the developed oral spray formulations was monitored during stability studies conducted under different storage conditions. Probiotic counts were determined at the beginning of the study and after 3 months of storage under refrigerated conditions (4°C ± 2°C), room temperature (25°C ± 2°C, 60% relative humidity), and accelerated conditions (40°C ± 2°C, 75% relative humidity). The results are presented in Table 4 and expressed as log cfu/mL (mean ± SD, *n* = 3). At the initial stage, all formulations contained approximately 9.00 log cfu/mL of viable probiotic cells. During the storage period, probiotic viability remained relatively stable under refrigerated conditions. After 3 months at 4°C ± 2°C, the viable counts ranged between 8.50 ± 0.09 and 8.62 ± 0.10 log cfu/mL among the formulations, indicating only a slight decrease compared with the initial value. Similarly, storage at 25°C ± 2°C and 60% relative humidity resulted in minimal reductions in probiotic viability. After 3 months, viable counts ranged from 8.30 ± 0.16 to 8.46 ± 0.13 log cfu/mL. These results demonstrate that the majority of the formulations were able to maintain probiotic levels above 10^8^ cfu/mL during storage at room temperature. Under accelerated stability conditions (40°C ± 2°C, 75% relative humidity), probiotic survival varied among the formulations. Formulations F2, F3, and F4 maintained relatively high probiotic counts, ranging from 8.12 ± 0.17 to 8.42 ± 0.14 log cfu/mL after 3 months. In contrast, formulation F1 exhibited a more pronounced decrease in viability, reaching 6.10 ± 0.18 log cfu/mL under the same conditions. The preservation of probiotic populations above 8 log cfu/mL in most formulations indicates that the developed formulation matrix provided an adequate protective environment for probiotic cells during storage. Maintaining probiotic levels above approximately 10^8^ cfu/mL is generally considered necessary for ensuring the functional efficacy of probiotic products. The relatively stable probiotic viability observed in this study may be attributed to the microencapsulation strategy used during formulation development. Encapsulation with maltodextrin and gum arabic has been widely reported to enhance the stability of probiotic cells by protecting them from environmental stresses such as temperature fluctuations, dehydration, and oxidative damage (Das et al. 2014). The polymeric matrix formed by these wall materials may act as a protective barrier that reduces cellular damage during storage. In addition, the presence of humectants such as glycerin and sorbitol 70% in the formulations may contribute to maintaining a stable microenvironment around probiotic cells, thereby reducing dehydration stress and preserving cell membrane integrity. Previous studies have shown that such excipients can improve probiotic survival during storage by stabilizing cellular structures and metabolic activity (Tripathi and Giri 2014). Overall, the results demonstrate that the developed oral spray formulations were able to maintain viable probiotic populations throughout the stability study, particularly under refrigerated and room temperature conditions. These findings indicate that the formulation system provides a suitable environment for preserving probiotic viability during storage and may therefore support the functional performance of the probiotic oral spray. Although probiotic release kinetics were not directly evaluated in artificial saliva within the scope of the present study, the maltodextrin–gum arabic microencapsulation system is expected to gradually hydrate and release viable probiotic cells upon contact with saliva. The hydrophilic nature of both wall materials facilitates water uptake and matrix dissolution, thereby enabling the release of entrapped microorganisms in the oral environment. Nevertheless, detailed release studies using simulated saliva are required to better characterize probiotic release behavior and retention within the oral cavity. In addition, the performance of the developed formulation under simulated oral conditions was not investigated in the present study. Factors such as artificial saliva exposure, salivary enzymes, continuous salivary flow, and repeated oral administration may influence probiotic survival, release behavior, and formulation retention within the oral cavity. Therefore, future studies should evaluate the stability and functionality of the formulation under simulated oral conditions to better predict its in vivo performance and clinical applicability. The three‐month stability period was selected as an initial real‐time and accelerated evaluation to determine whether the developed formulation could maintain acceptable physicochemical properties and probiotic viability under different storage conditions. Such preliminary stability studies are commonly employed during the early stages of formulation development to identify potential stability concerns before initiating longer‐term investigations. Although the results demonstrated satisfactory stability throughout the study period, the available data are not sufficient to establish a definitive shelf life. Therefore, extended stability studies (6–12 months) conducted under ICH‐relevant storage conditions will be required before commercial application and shelf‐life assignment can be considered. Although the 3‐month accelerated and real‐time stability studies provided preliminary evidence of formulation stability, longer‐term studies (6–12 months) will be required to fully establish shelf life and commercial applicability.

**TABLE 4:** Viability of *Lactiplantibacillus pentosus in* oral spray formulations during storage under different stability conditions (log cfu/mL).

Formulation	Initial Value (0 day)	4°C ± 2°C (3 months)	25°C ± 2°C, 60% RH (3 months)	40°C ± 2°C, 75% RH (3 months)
F1	9.00 (10^9^ cfu/mL)	8.51 ± 0.12	8.32 ± 0.15	6.10 ± 0.18
F2	9.00 (10^9^ cfu/mL)	8.62 ± 0.10	8.46 ± 0.13	8.42 ± 0.14
F3	9.00 (10^9^ cfu/mL)	8.58 ± 0.11	8.30 ± 0.16	8.12 ± 0.17
F4	9.00 (10^9^ cfu/mL)	8.50 ± 0.09	8.35 ± 0.12	8.30 ± 0.13

### 3.9 pH Measurement

**FIGURE 7:**
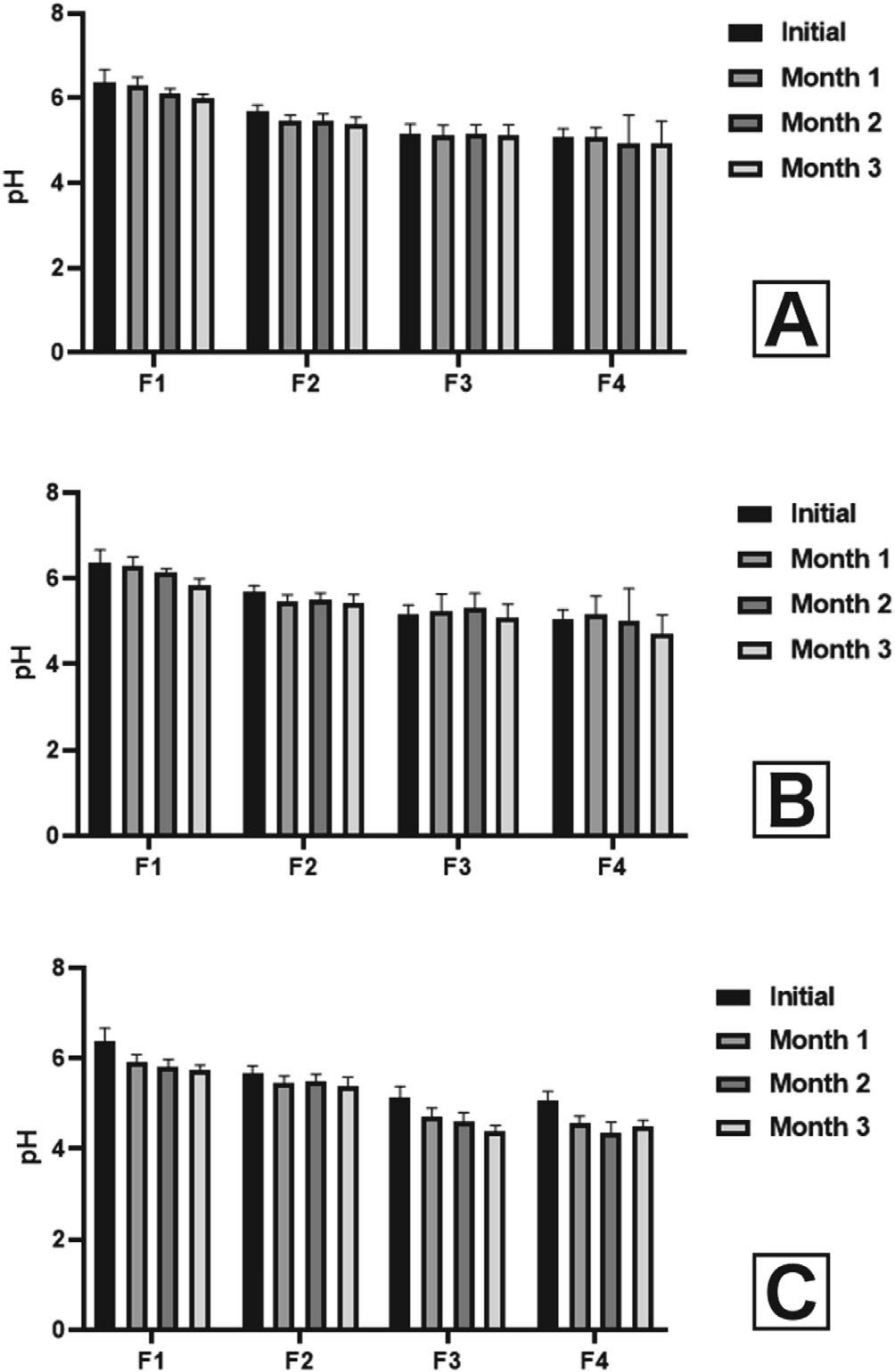
pH stability of the developed oral spray formulations during storage under different conditions. (A) Refrigerated storage (4°C ± 2°C), (B) Room temperature (25°C ± 2°C/60% ± 5% RH), and (C) Accelerated storage (40°C ± 2°C/75% ± 5% RH) over a period of 3 months. Data are expressed as mean ± SD (*n* = 3).

The pH values of the oral spray formulations were monitored during the 3‐month stability study under refrigerated (4°C), room temperature (25°C/60% RH), and accelerated (40°C/75% RH) storage conditions (Figure 7). At the initial time point, the formulations exhibited slightly acidic pH values ranging between approximately 5.0 and 6.4, which is considered suitable for oral mucosal applications and compatible with probiotic stability. Among the tested formulations, F1 showed the highest initial pH value (6.36 ± 0.30), while F3 and F4 exhibited comparatively lower pH values. Under refrigerated storage (4°C), only minor fluctuations were observed during the 3‐month period. The pH values of all formulations remained relatively stable, with changes generally remaining within 0.3–0.5 pH units. In particular, the pH of F1 decreased slightly from 6.36 ± 0.30 to 6.00 ± 0.10, indicating good stability of the formulation under cold storage conditions.

During storage at room temperature (25°C/60% RH), a similar trend was observed, with the pH values of all formulations showing modest decreases over time. For example, the pH of F1 gradually decreased from 6.36 ± 0.30 to 5.63 ± 0.15 after 3 months. Despite this slight decline, the pH values remained within a physiologically acceptable range for oral formulations. These slight variations may be attributed to minor interactions among formulation components, possible changes in ionic equilibrium, or gradual modifications in the aqueous phase during storage (Maslii et al. 2020). More pronounced pH reductions were observed under accelerated stability conditions (40°C/75% RH). For instance, the pH of F1 decreased from 6.36 ± 0.30 to 5.74 ± 0.12 after 3 months. Similar decreases were also observed for the other formulations. Elevated temperature and humidity are known to accelerate physicochemical processes such as component interactions, hydrolysis reactions, or shifts in ionic equilibrium, which may explain the observed pH reductions. Among the tested formulations, F1 demonstrated the most stable pH profile across all storage conditions, showing relatively smaller variations compared with the other formulations. In contrast, F3 and F4 exhibited slightly larger pH fluctuations, particularly under accelerated storage conditions. Overall, the results indicate that the formulations maintained acceptable pH stability throughout the study period, and the observed variations were relatively small and did not exceed the limits typically considered acceptable for liquid oral formulations. Such pH variations may also contribute to the rheological changes observed during storage, since even small pH shifts can influence intermolecular interactions and structural organization within liquid formulations. The superior pH stability of F1, together with its favorable rheological properties and stable organoleptic characteristics, supports its selection as the optimum formulation for further evaluation. Sodium bicarbonate was incorporated at a relatively low concentration (0.2%, w/v) primarily to provide buffering capacity and contribute to pH stabilization within the formulation. Throughout the stability study, the pH values remained within a range generally considered compatible with oral tissues. However, since enamel integrity and remineralization potential were not directly evaluated in the present study, the possible effects of the formulation on dental enamel cannot be conclusively determined. Future studies should therefore investigate enamel remineralization, demineralization, and buffering performance under simulated oral conditions to further establish the oral safety profile of the developed formulation.

### 3.10 Rheological Analysis

**FIGURE 8:**
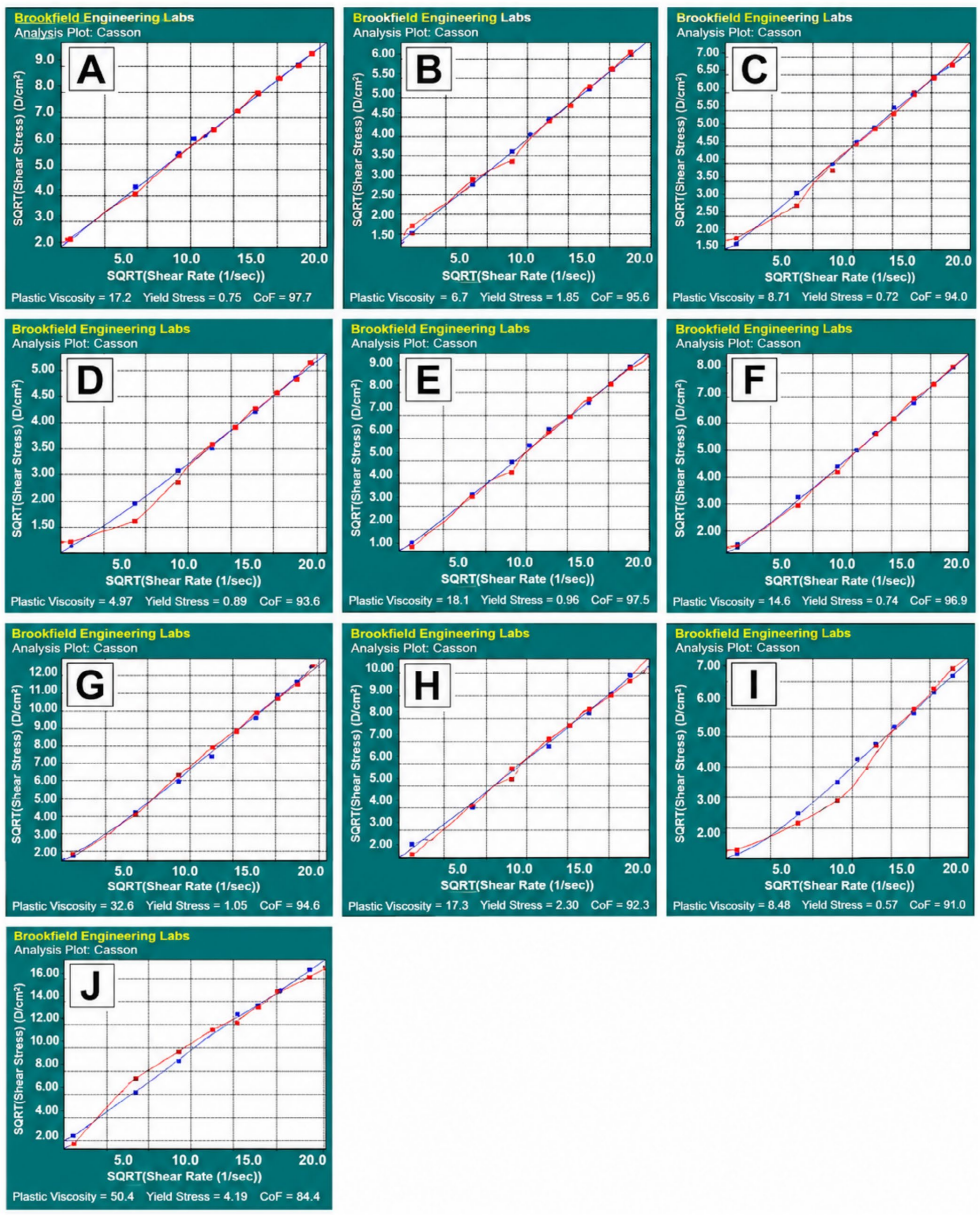
Casson rheograms of the F1 oral spray formulation obtained during stability studies under different storage conditions. Casson plots representing the rheological behavior of the F1 oral spray formulation during storage under refrigerated (4°C), room temperature (25°C/60% RH), and accelerated (40°C/75% RH) stability conditions. Plastic viscosity (PV), yield stress (YS), and coefficient of fit (COF) values were obtained from Casson model analysis. (A) Rheogram at the initial time (0 month) (B) Rheogram after 1 month of storage at 4°C (C) Rheogram after 2 months of storage at 4°C (D) Rheogram after 3 months of storage at 4°C (E) Rheogram after 1 month of storage at 25°C/60% RH (F) Rheogram after 2 months of storage at 25°C/60% RH (G) Rheogram after 3 months of storage at 25°C/60% RH (H) Rheogram after 1 month of storage at 40°C/75% RH (I) Rheogram after 2 months of storage at 40°C/75% RH (J) Rheogram after 3 months of storage at 40°C/75% RH. Data are expressed as mean ± SD (*n* = 3).

The rheological behavior of the F1 oral spray formulation during storage was evaluated using the Casson model, which is commonly employed to describe structured liquid systems exhibiting yield stress (Figure 8). In this model, the plastic viscosity (PV) represents the resistance of the system to flow once movement begins, while the yield stress (YS) indicates the minimum stress required to initiate flow. The coefficient of fit (COF) reflects the degree to which the experimental data conform to the Casson model, with values approaching 100 indicating excellent agreement between the experimental data and the Casson model (Abu‐Rumman et al. 2022; Šeremet et al. 2025; Vijayaratnam et al. 2015). At the initial time point (0 month), the formulation exhibited a PV of 17.2 and YS of 0.75, with a COF value of 97.7, indicating that the rheological behavior of the system was very well described by the Casson model. This suggests that the oral spray formulation possessed a moderately structured liquid character with a measurable yield stress, which can be advantageous for maintaining physical stability during storage while still allowing adequate flow during spray administration (Aidoo et al. 2015).

Under refrigerated conditions (4°C), the PV values decreased over time from the initial value of 17.2 to 6.17, 8.71, and 4.97 at the 1st, 2nd, and 3rd months, respectively. The YS values were measured as 1.85, 0.72, and 0.89, while the corresponding COF values slightly decreased from the initial value to 95.6, 94.0, and 93.6. Despite the reduction in PV, the COF values remained high, indicating that the flow behavior continued to conform well to the Casson model throughout storage. The decrease in PV may suggest a reduction in internal flow resistance, possibly due to minor structural rearrangements within the formulation at lower temperatures. However, since the YS values remained relatively close to the initial level after the first month, the formulation appears to have largely preserved its structured flow characteristics under refrigerated storage. For samples stored under room temperature conditions (25°C/60% RH), the PV values were 18.1, 14.6, and 32.6 for the 1st, 2nd, and 3rd months, respectively, while the YS values were 0.96, 0.74, and 1.05. The corresponding COF values remained high at 96.9, 97.5, and 94.6, indicating a strong agreement with the Casson model throughout the storage period. The relatively stable YS values suggest that the minimum stress required to initiate flow remained largely unchanged under these conditions. However, the increase in PV observed in the third month may indicate a gradual strengthening of internal interactions within the system, possibly due to slight changes in the dispersion structure or increased intermolecular interactions among formulation components. Nevertheless, the high COF values confirm that the overall flow behavior remained consistent with the Casson model, suggesting that the formulation maintained a stable rheological profile under these storage conditions. More pronounced changes were observed under accelerated stability conditions (40°C/75% RH). The PV values changed from 17.3 and 8.48 during the first and second months to a markedly higher value of 60.4 in the third month. Similarly, the YS values were 2.30, 0.57, and 4.9, indicating a significant increase by the third month. The COF values gradually decreased during storage, from 92.3 and 91.0 in the first 2 months to 84.4 in the third month. Although the Casson model still described the flow behavior reasonably well in the early stages of storage, the simultaneous increase in PV and YS accompanied by a decrease in COF suggests that substantial rheological changes occurred under accelerated conditions. The marked increase in both parameters indicates that the formulation became more resistant to flow and required greater stress to initiate movement, which may be attributed to structural rearrangements, possible solvent loss, or enhanced interactions among formulation components at elevated temperature and humidity. The results indicate that the F1 oral spray formulation maintained its Casson‐type flow behavior with high COF values under refrigerated and room temperature conditions, while only moderate variations in PV and YS were observed. In contrast, accelerated storage conditions led to more pronounced rheological changes, particularly after prolonged storage, suggesting that elevated temperature and humidity may significantly affect the internal structure and flow resistance of the formulation. Although sodium hyaluronate was incorporated because of its well‐documented mucoadhesive properties and its potential to prolong residence time on oral mucosal surfaces, direct evaluation of mucoadhesion was not performed in the present study. Consequently, the ability of the formulation to adhere to oral mucosa, resist salivary clearance, and maintain prolonged retention within the oral cavity could not be experimentally confirmed. Future studies should therefore include ex vivo or in vitro mucoadhesion testing, retention time measurements on oral mucosal models, and simulated saliva resistance studies to better characterize the mechanism of action and residence behavior of the developed oral spray.

### 3.11 Organoleptic Evaluation

Organoleptic evaluation showed no noticeable changes in taste, color, odor, or overall appearance of the formulations during storage (Table 5). Under refrigerated (4°C ± 2°C), room temperature (25°C ± 2°C/60% RH), and accelerated (40°C ± 2°C/75% RH) conditions, the formulations maintained their initial organoleptic properties throughout the 3‐month period. However, despite the stability of organoleptic characteristics, some variations were observed in pH and rheological parameters. Such changes may be attributed to minor structural rearrangements, intermolecular interactions among formulation components, or gradual modifications in the dispersion structure during storage. Nevertheless, these variations did not affect the observable physical characteristics of the formulations, indicating that the overall physical stability of the systems was maintained (Karimi et al. 2023; Pinto et al. 2021; Szymańska et al. 2018).

**TABLE 5:** Organoleptic properties of the formulations at the initial time and during the 3‐month stability study under different storage conditions.

Code	Taste				Color				Odor				Appearance			
	Initial	1 months	2 months	3 months	Initial	1 months	2 months	3 months	Initial	1 months	2 months	3 months	Initial	1 months	2 months	3 months
4°C ± 2°C																
F1	G	G	G	G	Y	Y	Y	Y	C	C	C	C	H	H	H	H
25°C ± 2°C/60% ± 5% RH																
F1	G	G	G	G	Y	Y	Y	Y	C	C	C	C	H	H	H	H
40°C ± 2°C/75% ± 5% RH																
F1	G	G	G	G	Y	Y	Y	Y	C	C	C	C	H	H	H	H

Abbreviations: C, Characteristic; G, Good; H, Homogeneous; Y, Yellow.

## 4 Conclusion

This study presents the development and comprehensive evaluation of a probiotic oral spray formulation designed to support oral microbial balance through a combined pharmabiotic approach. The microencapsulation of *Lactiplantibacillus pentosus* using maltodextrin and gum arabic successfully preserved probiotic viability and provided an effective strategy for improving formulation stability. The developed formulations demonstrated selective antibacterial activity against key Gram‐positive oral pathogens and showed substantial antibiofilm inhibition, indicating their potential to interfere with biofilm formation associated with dental plaque and oral infections. In addition to their antimicrobial effects, the formulations exhibited measurable antioxidant capacity, which may help reduce oxidative stress within the oral environment. Stability studies further confirmed that the formulation matrix was able to maintain probiotic viability at therapeutically relevant levels during storage, particularly under refrigerated and room temperature conditions. Among the tested formulations, F1 displayed the most balanced physicochemical properties and stability profile, making it the most suitable candidate for further development. Taken together, these findings suggest that the proposed probiotic oral spray system offers a promising platform for delivering beneficial microorganisms together with bioactive plant compounds in a stable and functional form. By integrating probiotic activity, antibiofilm potential, and antioxidant effects, this formulation may contribute to maintaining oral microbial homeostasis and preventing biofilm‐related oral disorders. Despite these promising findings, several limitations should be acknowledged. The present study was primarily focused on formulation development and in vitro characterization; therefore, the optimal dosage regimen, administration frequency, and duration of use were not established. The stability evaluation was limited to a three‐month period under refrigerated, room‐temperature, and accelerated storage conditions. Although the obtained results demonstrated satisfactory physicochemical stability and probiotic viability over the three‐month study period, longer‐term stability studies (6–12 months) conducted under a broader range of environmental conditions are required to fully establish shelf life and commercial applicability. Furthermore, the biological activities were evaluated against selected oral microorganisms and do not fully reflect the complexity of the oral microbiome ecosystem. Although the formulations demonstrated satisfactory physicochemical stability and probiotic viability over a three‐month storage period, longer‐term stability studies under a broader range of environmental conditions are required. In addition, consumer‐oriented sensory acceptance studies involving trained or untrained volunteers should be conducted in future investigations to evaluate taste perception, mouthfeel, ease of administration, and overall product acceptability, which are important factors for long‐term patient compliance. The future studies should investigate the behavior of the formulation under simulated oral conditions, including artificial saliva exposure, salivary flow, and repeated administration models. The organoleptic evaluation performed in the present study was intended as a preliminary assessment of formulation stability and did not include formal sensory acceptance testing. Future studies should incorporate trained sensory panels or consumer‐based evaluations to provide a more comprehensive assessment of taste, odor, mouthfeel, and overall product acceptability. Future investigations should also include in vivo studies and well‐designed clinical trials to determine optimal dosing strategies, confirm safety and efficacy in human subjects, evaluate potential effects on enamel integrity and oral microbial ecology, and validate the long‐term therapeutic potential of the developed pharmabiotic oral spray.

## Author Contributions

**A. Alper Öztürk:** validation, writing – review and editing, formal analysis, supervision, investigation. **Pervin Soyer:** conceptualization, investigation, writing – original draft, methodology, validation, writing – review and editing, formal analysis.

## Funding

The authors have nothing to report.

## Ethics Statement

The authors have nothing to report.

## Conflicts of Interest

The authors declare no conflicts of interest.

## Data Availability

The data that support the findings of this study are available from the corresponding author upon reasonable request.
